# Nanomedicine‐Enabled Photonic Thermogaseous Cancer Therapy

**DOI:** 10.1002/advs.201901954

**Published:** 2019-11-26

**Authors:** Haohao Yin, Xin Guan, Han Lin, Yinying Pu, Yan Fang, Wenwen Yue, Bangguo Zhou, Qiao Wang, Yu Chen, Huixiong Xu

**Affiliations:** ^1^ Department of Medical Ultrasound Shanghai Tenth People's Hospital Tongji University School of Medicine Shanghai 200072 P. R. China; ^2^ State Key Laboratory of High Performance Ceramics and Superfine Microstructure Shanghai Institute of Ceramics Chinese Academy of Sciences Shanghai 200050 P. R. China

**Keywords:** MXene, nitric oxide, photonic nanomedicine, sequential activation, thermogaseous therapy

## Abstract

Local photothermal hyperthermia for tumor ablation and specific stimuliresponsive gas therapy feature the merits of remote operation, noninvasive intervention, and in situ tumor‐specific activation in cancer‐therapeutic biomedicine. Inspired by synergistic/sequential therapeutic modality, herein a novel therapeutic modality is reported based on the construction of two‐dimensional (2D) core/shell‐structured Nb_2_C–MSNs–SNO composite nanosheets for photonic thermogaseous therapy. A phototriggered thermogas‐generating nanoreactor is designed via mesoporous silica layer coating on the surface of Nb_2_C MXene nanosheets, where the mesopores provide the reservoirs for NO donor (S‐nitrosothiol (RSNO)), and the core of Nb_2_C produces heat shock upon second near‐infrared biowindow (NIR‐II) laser irradiation. The Nb_2_C–MSNs–SNO‐enabled photonic thermogaseous therapy undergoes a sequential process of phototriggered heat production from the core of Nb_2_C and thermotriggered NO generation, together with photoacoustic‐imaging (PAI) guidance and monitoring. The constructed Nb_2_C–MSNs–SNO nanoreactors exhibit high‐NIR‐induced photothermal effect, intense NIR‐controlled NO release, and desirable PAI performance. Based on these unique theranostic properties of Nb_2_C–MSNs–SNO nanocomposites, sequential photonic thermogaseous therapy with limited systematic toxicity on efficiently suppressing tumor growth is achieved by PAI‐guided NIR‐controlled NO release as well as heat generation. Such a thermogaseous approach representes a stimuli‐selective strategy for synergistic/sequential cancer treatment.

## Graphical abstract

A noninvasive tumor‐therapy modality based on a two‐dimensional (2D) core/shell‐structured nanoreactor (Nb_2_C–MSNs–SNO composite nanosheets) is established, i.e., photonic thermogaseous cancer therapy. Once irradiated by a near‐infrared biowindow (NIR‐II) laser, the “core” of this nanomedicine can rapidly produce thermal shock, which will further sequentially achieve the controllable NO generation for thermogaseous therapy.

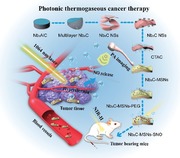

## 1 Introduction

The integration of various therapeutic strategies by synergistic enhancement, sequential process, and cascade amplification effect has achieved great promotions in oncotherapy, such as cocktail strategies,^1^ photothermal therapy (PTT)/chemotherapy,^2^ photodynamic therapy (PDT)/immunotherapy,^3^ sonodynamic therapy (SDT)/chemotherapy,^4^ gas therapy/PDT,^5^ and gas therapy/ultrasound therapy.^6^ Benefit from these therapeutic integrations, progressive outcomes have been achieved, including specific targeting to cancer cell by proliferation cycle or divergent metabolic pathways, reducing the toxicity and side effects from drugs, inhibiting multidrug resistant (MDR) of cancer cells, and crossing the blood–brain barrier (BBB).^7^ Gas therapy, an emerging therapeutic modality, has received great attention very recently by employing diverse gasotransmitters to induce apoptosis of tumor tissues.^8^ To date, the general gas gasotransmitters applied in gas therapy involve hydrogen sulfide (H_2_S),^9^ carbon monoxide(CO),^10^ hydrogen (H_2_),^11^ and nitric oxide (NO).^7^ As an emerging molecule of theranostic gas, NO plays an indispensable character in versatile physiological and pathological activities, including wound healing and regulation of vascular smooth muscle and neurons.^12^ Interestingly, high‐content NO (>1 µmol) not only eliminates tumor cells through various ways, including oxidative and nitrosative stress, damage of mitochondria and DNA, and inhibition of cellular repair, but also synergistically enhances other therapeutics, such as chemotherapy, radiotherapy, and PDT.^13^ It has been reported that the clearance effect of NO by cancer cells is closely related to its concentration and release rate.^14^ However, NO molecules feature a short half‐life in vivo and is prone to react with bio‐macromolecules and free radicals, so it is difficult for NO to achieve effective enrichment in tumor region. Therefore, developing external‐responsive and local‐triggered NO donors can be employed for on‐demand NO release. Compared to other NO donors, S‐nitrosothiol (RSNO) has a unique advantage of high biocompatibility.^15^ RSNO can release NO through a variety of stimuli, including transition metals (such as Cu^+^),^16^ ascorbic acid,^17^ ultraviolet light,^18^ heat,^19^ or enzymes (superoxide dismutase and protein disulfide isomerase).^20^ For instance, under the heat shock, the S—NO bond in RSNO is split to generate NO.^21^

Photonic hyperthermia such as PTT for oncotherapy, depending on photothermal agents (PTAs) to in situ generate heat under near‐infrared (NIR) laser radiation for tumor eradication, has been well explored and attracted in‐depth attention.^22^ Compared with conventional therapeutic modalities, PTT features remote and noninvasive therapeutic characteristics with minimized damage to normal tissue by its instinct high temporal/spatial monitoring of local temperature.^23^ In comparison to endogenous pH/enzyme triggers, NIR is featured with unique advantages in triggering drug release as an exogenous stimulus.^24^ First, the on/off drug‐releasing behavior at a specific focal site is based on the precise irradiation of NIR laser. Second, the on‐demand drug releasing/triggering can be controlled by a facile regulation on NIR output energy. Meanwhile, as PTAs, 2D nanosheets induced by NIR irradiation have attracted extensive attention for tumor ablation in recent years, such as graphene,^25^ MoS_2_,^26^ palladium (Pd) nanosheets,^27^ and black phosphorus (BP).^28^ Especially, MXenes^29^ represent an emerging multifunctional 2D solid crystals containing plenty of transition metal carbides and carbonitrides with thermal conductivity and hydrophilic nature, as well as excellent properties for photothermal conversion and drug delivery. Moreover, as a wide‐spectrum photothermal‐conversion agent, MXenes can be activated by laser irradiation in either the first NIR biowindow (NIR‐I) (750–1000 nm) or the second NIR biowindow (NIR‐II) (1000–1350 nm).^30^, ^31^ The NIR‐II exhibits larger penetration depth and higher maximum permissible exposure (MPE) for skin than NIR‐I, where the MPE values of NIR‐I and NIR‐II are 1 and 0.33 W cm^−2^, respectively.^32^ Inspired by the fact that MXene has superior photothermal properties and NO donor of RSNO that can generate NO under hyperthermia shock, combining the properties of these two therapeutic modalities could achieve a synergistic/sequential therapy process, in which photonic energy is converted into thermal energy, further promoting the release of NO for gas therapy. Therefore, exploitation for a photo‐triggered thermogas‐generating nanoreactor enabled by a sequential process, step‐by‐step photothermal conversion, and thermogas generation, can potentially fulfill the crucial prerequisites in cancer therapy.

**Scheme 1:**
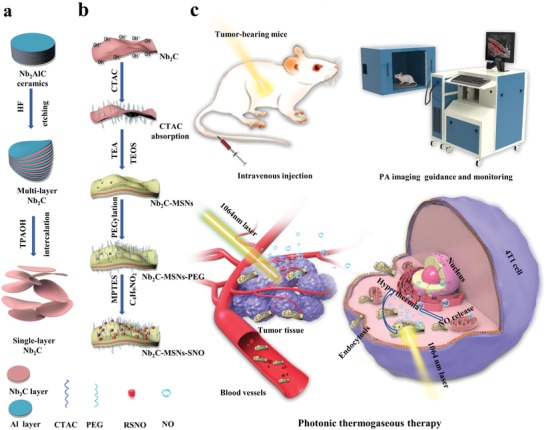
Schematic illustration of the synthetic process of 2D composite nanosheets Nb_2_C–MSNs–SNO and photonic thermogaseous therapy against cancer with potential PA‐imaging (PAI) guidance and monitoring. a) Scheme of the fabrication process of ultrathin Nb_2_C nanosheets by HF etching and TPAOH intercalation. b) Schematic diagram for the fabrication of Nb_2_C–MSNs–SNO, including CTAC absorption, mesoporous silica coating, PEGylation, and NO donor conjugation. c) Schematic illustration of theranostic functions of Nb_2_C–MSNs–SNO, including free delivery within blood vessel after intravenous injection, photothermal‐triggered NO release, photonic thermogaseous therapy toward oncotherapy, and PAI guidance and monitoring.

In this work, we construct a multifunctional Nb_2_C–MSNs–SNO composite nanosheet with mesoporous structure based on 2D Nb_2_C MXene for NIR‐II‐controlled NO generation, achieving sequentially synergistic photonic thermogaseous therapy, together with photoacoustic (PA) imaging (PAI) functionality (**Scheme** 1). In brief, through facile sol–gel chemistry, a mesoporous silica layer was uniformly deposited onto the surface of Nb_2_C MXene nanosheets, which improved the biocompatibility of MXene nanosheets and enriched the chemical composition for further surface modification and loading of the NO donor. As expected, such Nb_2_C–MSNs–SNO composite nanosheets not only exhibited high photothermal properties in vitro and in vivo, but also sequentially achieved the controllable NO generation by thermal shock. In addition, both in vitro and in vivo evaluations demonstrated that these Nb_2_C–MSNs–SNO composite nanosheets had excellent PA‐imaging effect, which was conducive to the potential real‐time monitoring of the whole therapeutic process to achieve accurate noninvasive treatment. Therefore, these multifunctional 2D composite nanosheets, by integrating the features of each component, would serve as a desirable nanomedicine to achieve photonic‐triggered thermogaseous therapy toward synergistic oncotherapy, which is expected to provide a new efficient cancer‐therapeutic modality.

## 2 Results and Discussion

### 2.1 Synthesis and Characterization of Nb_2_C–MSNs–SNO Composite Nanosheets

**Figure 1:**
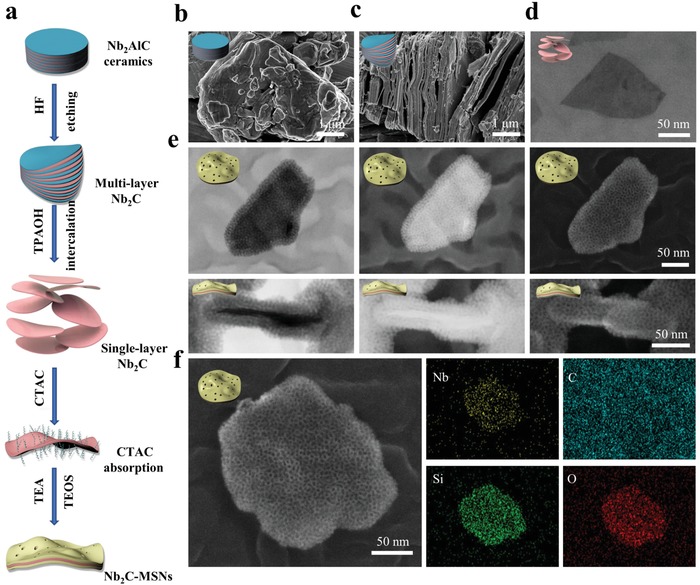
Synthesis and characterization of Nb_2_C–MSNs composite nanosheets. a) Schematic illustration showing the synthetic process of Nb_2_C–MSNs composite nanosheets. b–d) SEM images of: b) Nb_2_AlC ceramics, c) multilayer Nb_2_C after HF etching, and d) single‐layer Nb_2_C nanosheets after TPAOH intercalation. e) Bright‐field TEM (left), dark‐field TEM (middle), and SEM (right) images of Nb_2_C–MSNs (vertical (down) and lateral (upper) views, scale bar: 50 nm). f) SEM image and corresponding elemental mapping of Nb_2_C–MSNs composite nanosheets (Nb, C, Si, and O; scale bar: 50 nm).

The synthesis of 2D core/shell‐structured Nb_2_C–MSNs based on initially synthesized Nb_2_C MXene was achieved by a typical sol–gel approach for mesoporous silica layer coating (**Figure** 1a). First, ultrathin Nb_2_C nanosheets were fabricated by a wet‐chemical exfoliation strategy according to our previous report.29a To remove the Al layer, 40% HF aqueous was applied to etch the Nb_2_AlC bulk precursor. Then, the resulting multilayered, accordion‐like Nb_2_C nanosheets were intercalated in tetrapropylammonium hydroxide (TPAOH) aqueous to obtain ultrathin few‐layer Nb_2_C nanosheets. Scanning electron microscopy (SEM) images and corresponding elemental mapping show that Nb_2_AlC bulk exhibits the typical layered ternary compound containing elements of Nb, Al, and C (Figure 1b; Figure S1a, Supporting Information), and the microstructure of etched Nb_2_C powder exhibits well‐stacked and uniform nanosheets (Figure 1c; Figure S1b, Supporting Information). After further TPAOH intercalation, SEM images and corresponding elemental mapping reveal ultrathin Nb_2_C nanosheets with an average lateral size of ≈200 nm (Figure 1d). It has been verified that the surface of MXene is present with abundant —OH group, based on which the surface of Nb_2_C nanosheets was further absorbed with cetyltrimethylammonium chloride (CTAC) solution (as the mesopore‐making and structure‐directing agent) via electrostatic interaction.^30^ In addition, tetraethyl orthosilicate (TEOS), as the silica source, was introduced into reaction system to form mesoporous silica layer on the surface of ultrathin Nb_2_C nanosheets by hydrolyzed/condensed silicon oligomers and their further self‐assembly with CTAC (designated as Nb_2_C–MSNs). Finally, methanol/sodium chloride mixture solution was employed to extract the CTAC surfactants. Transmission electron microscope (TEM) images exhibit that the surface of Nb_2_C were covered with uniform mesoporous silica layer (Figure S2, Supporting Information).

**Figure 2:**
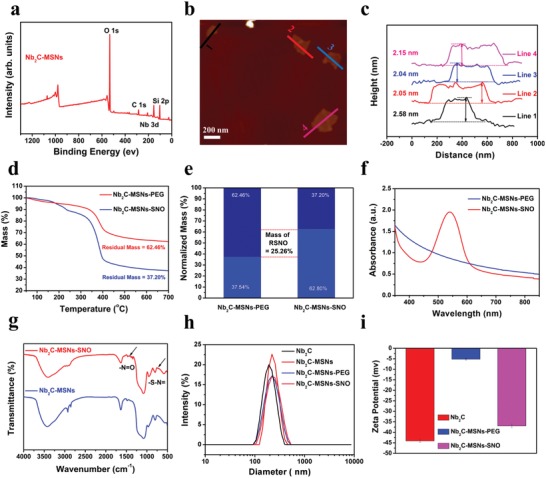
Characterization of Nb_2_C–MSNs and Nb_2_C–MSNs–SNO. a) XPS spectrum of Nb_2_C–MSNs composite nanosheets. b) AFM image and c) corresponding thickness distribution of Nb_2_C–MSNs composite nanosheets. d) Thermogravimetric analysis (TGA) and e) normalized weight loss distribution diagram of Nb_2_C–MSNs–PEG and Nb_2_C–MSNs–SNO. f) UV–vis–NIR spectra of Nb_2_C–MSNs–PEG and Nb_2_C–MSNs–SNO after added into the Griess agents. g) FTIR spectra of Nb_2_C–MSNs and Nb_2_C–MSNs–SNO. h) Particle‐size distribution of Nb_2_C, Nb_2_C–MSNs, Nb_2_C–MSNs–PEG, and Nb_2_C–MSNs–SNO. i) Zeta potential of Nb_2_C, Nb_2_C–MSNs–PEG, and Nb_2_C–MSNs–SNO.

Additionally, TEM images, secondary electron images, and elemental mapping obviously show the sandwich structure and composition of Nb_2_C–MSNs nanosheets, demonstrating the formation of desirable mesoporous structure, 2D planar topology, and the planar distribution of Si/O element in Nb_2_C–MSNs composite nanosheets (Figure 1e,f; Figure S1c, Supporting Information). Abundant mesopores of Nb_2_C–MSNs made molecules diffusion, loading, and releasing possible. X‐ray photoelectron spectroscopy (XPS) was used to analyze the elemental status of Nb_2_C–MSNs composite nanosheets. The XPS survey spectrum shows the existence of Nb, C, Si, and O elements (**Figure** 2a; Figure S3a–d, Supporting Information). After the surface engineering of Nb_2_C with mesoporous silica, an emerging peak of Si appeared in the XPS survey spectrum of Nb_2_C–MSNs. The characteristic peaks at 103.5 and 104.1 eV in the Si 2p spectrum were indexed to Si—O bond of mesoporous silica layer, which is consistent with the previous report (Figure S3e,f, Supporting Information).^30^ The atomic force microscopy (AFM) further demonstrates that the initial Nb_2_C nanosheets were coated with mesoporous silica layer (Figure 2b). The thickness of Nb_2_C–MSNs (≈2 nm) was measured by AFM. The enlarged thickness was attributed to mesoporous silica coating (Figure 2c). The lateral size of Nb_2_C and Nb_2_C–MSNs was determined to be ≈200 nm by AFM, which matched the result of TEM characterization (Figure S4, Supporting Information).

Benefiting from the uniform mesoporous silica layer on the surface Nb_2_C MXene to provide reservoirs for guest molecules, the NO donor was encapsulated into the mesopores of Nb_2_C–MSNs composite nanosheets. To improve stability of Nb_2_C–MSNs under physiological conditions, methoxyl‐poly(ethylene glycol)‐silane (mPEG–silane) was further modified onto the surface of Nb_2_C–MSNs (designated as Nb_2_C–MSNs–PEG). In order to graft RSNO, NO donor, into mesoporous silica, Nb_2_C–MSNs–PEG was initially modified with —SH group and then reacted with *tert*‐butyl nitrite (designated as Nb_2_C–MSNs–SNO). The loading capacity of the NO donor was investigated by thermogravimetric analysis (TGA; Figure 2d), from which the loading amount of RSNO was determined to be as high as 25.26% (Figure 2e), presenting the potential high efficiency of thermogaseous therapy. In order to further determine the successful loading of NO the donor within the mesoporous structure of Nb_2_C–MSNs–PEG, UV–vis–NIR spectra of Nb_2_C–MSNs–PEG and Nb_2_C–MSNs–SNO were detected. After the addition of Griess reagent, it could be found that a strong peak at 540 nm appeared in Nb_2_C–MSNs–SNO rather than Nb_2_C–MSNs–PEG, demonstrating the successful loading of the NO donor within Nb_2_C–MSNs–PEG (Figure 2f). In addition, the Fourier transform infrared spectroscopy (FTIR) spectrum of Nb_2_C–MSNs–SNO shows two emerging characteristic peaks of —N=O and —S—N= at 1467 and 723 cm^−1^, respectively (Figure 2g), which confirms the successful conjugation of —SNO into Nb_2_C–MSNs–PEG.^7^

Dynamic light scattering (DLS) was utilized to measure the average hydrodynamic diameters of Nb_2_C, Nb_2_C–MSNs, Nb_2_C–MSNs–PEG, and Nb_2_C–MSNs–SNO, which were around 201, 229, 232, and 243 nm, respectively (Figure 2h). The increasing sizes were derived from the surface engineering of mesoporous silica shell, modification with mPEG–silane molecules, and conjugation of the NO donor. A series of zeta potential changes provide evidence for successive conjugation of mesoporous silica layer, PEG, and NO donor (Figure 2i). Owing to the coating of mesoporous silica on Nb_2_C surface and subsequent mPEG–silane modification, the surface potential of Nb_2_C increased from −44 to −5.2 mV, which also proved the successful mPEG–silane modification. Due to the negative potential of the NO donor, such a change on surface potential further suggested the successful loading of NO donor into mesoporous structure of Nb_2_C–MSNs–PEG composite nanosheets. The intrinsic negative surface potential of NO donor (RSNO) led the surface potential of Nb_2_C–MSNs–SNO decreasing to a more negative value, which further confirms the effective loading of the NO donor. In addition, owing to the steric hindrance of organic chains, Nb_2_C–MSNs–SNO composite nanosheets are featured with excellent stability in different physiological solutions, including 0.9% aqueous NaCl (saline), phosphate‐buffered saline (PBS), fetal bovine serum (FBS), and Dulbecco's modified Eagle medium (DMEM; Figure S5a,b, Supporting Information). So far, the degradation property of inorganic nanoparticles is still an important issue that hinders their clinical transformation. Therefore, it is necessary to evaluate the degradation behavior of Nb_2_C–MSNs–SNO composite nanosheets in vitro. It has been found that the degradation was evident as indicated by the substantial shape changes in 5 days biodegradation treatment, and the original morphology of Nb_2_C–MSNs–SNO was almost completely disrupted and only very few sheet‐like objects could be observed in 7 days (Figure S6, Supporting Information). These results indicate that Nb_2_C–MSNs–SNO composite nanosheets could be gradually degraded under physiological conditions, laying a foundation for their biomedical applications.

### 2.2 NO Generation by NIR‐II Irradiation

**Figure 3:**
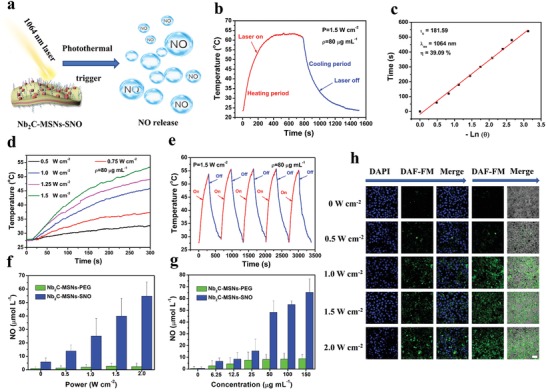
In vitro photothermal‐conversion performance and photothermal‐triggered NO release from Nb_2_C–MSNs–SNO composite nanosheets. a) Schematic illustration of NO release from Nb_2_C–MSNs–SNO under NIR‐II (1064 nm) laser irradiation. b) Photothermal profile of Nb_2_C–MSNs–SNO composite nanosheets dispersed suspension ([Nb] = 80 mg mL^−1^) under NIR‐II laser irradiation at 1.5 W cm^−2^ until reaching the steady‐state temperature and a cooling period after the laser was shut off. c) Time constant for heat transfer from the system by using the linear time data from the cooling period versus. d) Photothermal‐heating curves of Nb_2_C–MSNs–SNO at elevated power densities under 1064 nm laser irradiation ([Nb] = 80 µg mL^−1^). e) Recycling heating curves of Nb_2_C–MSNs–SNO suspension dispersed in deionized water with 1064 nm laser irradiation for five laser on/off cycles ([Nb] = 80 µg mL^−1^, 1.5 W cm^−2^). f,g) Quantitative assessment of NO release from Nb_2_C–MSNs–SNO and Nb_2_C–MSNs–PEG under varied treatments including: f) different power densities (0, 0.5, 1.0, 1.5, and 2.0 W cm^−1^, [Nb] = 50 µg mL^−1^) and g) varied concentrations (1.5 W cm^−2^, [Nb] = 0, 6.25, 12.5, 25, 50, 100, and 150 µg mL^−1^). h) Intracellular detection of NO release in 4T1 cancer cells after the coincubation with Nb_2_C–MSNs–SNO followed by laser irradiation at different power densities (0, 0.5, 1.0, 1.5, and 2.0 W cm^−2^, [Nb] = 50 µg mL^−1^; scale bar: 50 µm).

We then evaluated photonic thermogaseous NO‐generation behavior of Nb_2_C–MSNs–SNO composite nanosheets under NIR‐II irradiation (**Figure** 3a). First, to verify the potential photothermal‐conversion capability of Nb_2_C–MSNs–SNO composite nanosheets in NIR‐II, UV–vis–NIR spectra were recorded at varied concentrations ([Nb] = 1.25, 2,5, 5, 10, 20, and 40 µg mL^−1^), which show the characteristic absorption in the NIR‐II biowindow (Figure S7a, Supporting Information) with an extinction coefficient (ε) of 33.35 L g^−1^ cm^−1^ (Figure S7b, Supporting Information) according to Lambert–Beer law, proving that Nb_2_C–MSNs–SNO could act as a desirable candidate for photothermal conversion. Another key parameter of photothermal‐conversion performance, photothermal‐conversion efficiency (η), was calculated to be 39.09% (Figure 3b,c). These two key parameters demonstrate that Nb_2_C–MSNs–SNO composite nanosheets retain excellent photothermal characteristics derived from 2D Nb_2_C MXene. The in vitro photothermal transduction effect of Nb_2_C–MSNs–SNO was investigated at varied concentrations and NIR‐II power densities. First, the Nb_2_C–MSNs–SNO nanosheets with varied concentrations ([Nb] = 0, 10, 20, 40, 80, 160, and 320 µg mL^−1^) were irradiated by a 1064 nm laser of 1.0 W cm^−2^ (Figure S5d,e, Supporting Information). The temperature reached as high as 59.6 °C at the Nb concentration of 320 µg mL^−1^. However, the temperature of water only increased to 28.7 °C, which indicates that NIR‐II irradiation could be efficiently and rapidly converted into thermal energy in the presence of Nb_2_C–MSNs–SNO composite nanosheets. Afterward, Nb_2_C–MSNs–SNO composite nanosheets ([Nb] = 80 µg mL^−1^) were irradiated with NIR‐II laser for 5 min at varied power densities (0.5, 0.75, 1.0, 1.25, and 1.5 W cm^−2^) (Figure 3d). The temperature increased to 54.8 °C ([Nb] = 80 µg mL^−1^) under the exposure of 1.5 W cm^−2^. Above results demonstrate that a desired temperature elevation can be achieved by regulating concentration and NIR‐II irradiation energy. In addition, these Nb_2_C–MSNs–SNO composite nanosheets are featured with high photothermal stability during five heating/cooling cycles (Figure 3e), highlighting that such composite nanosheets could serve as desired photothermal agent for the following sequential gas generation during photonic thermogaseous therapy.

Then the standard Griess assay was applied to qualitatively measure the NO release from Nb_2_C–MSNs–SNO composite nanosheets. After Nb_2_C–MSNs–PEG and Nb_2_C–MSNs–SNO solutions were irradiated with 1064 nm laser for 10 min, the color of Nb_2_C–MSNs–SNO solution turned from black to red after adding the Griess reagent, which was in consistence with the positive control group (NaNO_2_) (Figure S8, Supporting Information). NIR‐II irradiation could effectively trigger NO release from Nb_2_C–MSNs–SNO. Quantitative investigation of the NO release from the composite nanosheets was also conducted based on Griess assay. The as‐prepared Nb_2_C–MSNs–PEG and Nb_2_C–MSNs–SNO solutions ([Nb] = 50 µg mL^−1^) were irradiated with 1064 nm laser at varied power densities (0, 0.5, 1.0, 1.5, and 2.0 W cm^−1^). Despite Nb_2_C–MSNs–SNO could spontaneously release a small amount of NO molecules, the 1064 nm laser irradiation could substantially accelerate the release of NO from Nb_2_C–MSNs–SNO, and the amount of NO increased as the laser power density elevated (Figure 3f).

In addition, the amount of NO release increased with the elevated concentrations of Nb_2_C–MSNs–SNO ([Nb] = 0, 6.25, 12.5, 25, 50, 100, and 150 µg mL^−1^) under NIR‐II irradiation (1.5 W cm^−2^), indicating the concentration‐dependent and NIR power density‐dependent features of NO releasing (Figure 3g). As a control, there was no NO generation regardless of increasing the Nb_2_C–MSNs–PEG concentration or elevating the NIR‐II power density, indicating that NO released from the cleavage of S=N bond in Nb_2_C–MSNs–SNO. Above results demonstrate that the photonic‐responsive “on‐demand” NO releasing behavior could be achieved by controlling the amount of the Nb_2_C–MSNs–SNO and the energy output of NIR‐II irradiation. Furthermore, commercial NO tracking agent, 3‐amino‐4‐aminomethyl‐2′,7′‐difluorescein, diacetate (DAF‐FM), was applied to track the intracellular NO generation (Figure 3h). The confocal laser scanning microscopy (CLSM) images show that a large amount of NO was released within 4T1 cancer cell when treated with 1064 nm laser irradiation, and the released NO amount increased with the elevated NIR‐II power density, indicating that Nb_2_C–MSNs–SNO ([Nb] = 50 µg mL^−1^) could release NO in situ under 1064 nm laser irradiation and the amount of NO could be well controlled by regulating the NIR‐II power density.

### 2.3 In Vitro Intracellular Endocytosis and Synergistic Therapy against 4T1 Cancer Cells

**Figure 4:**
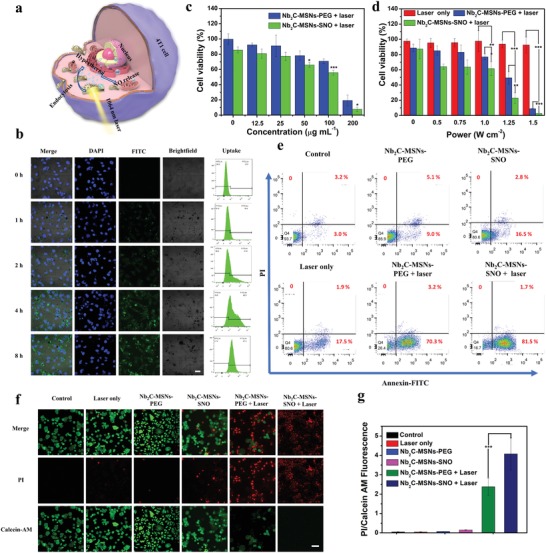
In vitro synergistic thermogaseous therapy. a) Schematic illustration of synergistic thermogaseous therapy against 4T1 cancer cells enabled by Nb_2_C–MSNs–SNO at the cell level. b) CLSM imaging and flow cytometry analysis of 4T1 cells incubated with FITC‐labeled Nb_2_C–MSNs–SNO for 0, 1, 2, 4, and 8 h. Scale bar: 20 µm. c,d) Cytotoxicity of Nb_2_C–MSNs–SNO and Nb_2_C–MSNs–PEG to 4T1 cells under different treatments including: c) different concentrations ([Nb] = 0, 12.5, 25, 50, 100, and 200 µg mL^−1^) and d) different power densities (0, 0.5, 0.75, 1.0, 1.25, and 1.5 W cm^−2^). (*P*‐values: * < 0.05, ** < 0.01, *** < 0.001). e) Flow cytometry quantification assay on relative viabilities of 4T1 cell after different treatments (PBS, Nb_2_C–MSNs–PEG, Nb_2_C–MSNs–SNO, 1064 nm laser only, Nb_2_C–MSNs–PEG + 1064 nm laser, and Nb_2_C–MSNs–SNO + 1064 nm laser). f) CLSM images of 4T1 cells stained by calcein AM (green) and PI (red) after different treatments. Scale bar: 50 µm. g) The ratio of PI/calcein AM of 4T1 cells after different treatments. (*P*‐values: * < 0.05, ** < 0.01, and *** < 0.001).

Encouraged by the desirable in vitro photothermal‐conversion performance and “on‐demand” NO release, sequential synergistic efficacy of thermogaseous therapy by Nb_2_C–MSNs–SNO was further investigated against 4T1 breast cancer cell line. Once the Nb_2_C–MSNs–SNO composite nanosheets endocytose into tumor cells, the core of Nb_2_C will be activated for photothermal conversion under NIR‐II irradiation, which can accelerate the release of NO from the mesopores to further achieve thermogaseous therapy (**Figure** 4a). The capacity of Nb_2_C–MSNs–SNO nanosheets to penetrate the cell membrane and enter the cytoplasm was initially investigated by CLSM. The CLSM images and flow cytometry analysis results show that the uptake process was time dependent, indicating that fluoresceine isothiocyanate (FITC)‐labeled Nb_2_C–MSNs–SNO nanosheets could be effectively uptaken by cancer cells (Figure 4b). In addition, in order to confirm that the Nb_2_C–MSNs–SNO nanosheets could enter the cell, the mechanism of cellular uptake was further investigated in detail. Three endocytosis inhibitors—sucrose, methyl‐β‐cyclodextrin (MβCD), and amiloride—were employed to verify the clathrin‐mediated endocytosis, caveolae‐mediated endocytosis, and micro‐pinocytosis, respectively. The CLSM images and flow cytometry analysis results indicate that the endocytosis efficiency significantly decreased in cells pretreated with MβCD and amiloride, suggesting that the endocytic uptake of Nb_2_C–MSNs–SNO composite nanosheets was mainly through the pathways of caveolae‐dependent endocytosis and micro‐pinocytosis (Figure S9, Supporting Information).

4T1 and human umbilical vein endothelial cell (HUVEC) cell lines were employed to investigate the cytotoxicity of Nb_2_C–MSNs–SNO (Figure S10, Supporting Information). It has been revealed that Nb_2_C–MSNs–SNO shows negligible toxicity to these two cell lines. The viabilities were above 85% even at a high concentration of 200 µg mL^−1^, demonstrating high biocompatibility of Nb_2_C–MSNs–SNO. Synergistic efficacy of photonic thermogaseous therapy of Nb_2_C–MSNs–SNO was assessed against 4T1 cancer cells by following treatments: varied concentration of composite nanosheets ([Nb] = 0, 12.5, 25, 50, 100, and 200 µg mL^−1^) and varied laser power densities (0, 0.5, 0.75, 1.0, 1.25, and 1.5 W cm^−2^) under NIR‐II laser irradiation. With the increase of Nb_2_C–MSNs–SNO concentrations and laser power densities, the cell viability gradually decreased (Figure 4c,d). Importantly, Nb_2_C–MSNs–SNO composite nanosheets showed enhanced cancer cell killing effect compared with that of Nb_2_C–MSNs–PEG composite nanosheets, indicating the high therapeutic efficacy of synergistic photonic thermogaseous therapeutic modality, especially at the laser power density of 1.25 W cm^−2^. The cell viability was 22% for the Nb_2_C–MSNs–SNO group, while nearly 50% cells were survival in Nb_2_C–MSNs–PEG group, which suggests that the NO could further induce apoptosis of cancer cells.

In addition, similar therapeutic results were observed by flow cytometry analysis (Figure 4e; Figure S11, Supporting Information). The Nb_2_C–MSNs–SNO under NIR‐II laser irradiation group at the power density of 1.5 W cm^−2^ exhibited obvious cell apoptosis (81.5%) compared with Nb_2_C–MSNs–PEG group (70.3%). These results confirm that the synergistic photonic thermogaseous therapy can efficiently induce cancer cell apoptosis. Living and dead 4T1 cells were further observed by Calcein‐AM (green, live cells)/propidium iodide (PI) (red, dead cells) fluorescence assay. The CLSM images showed strong green fluorescence signals in the control, laser only, Nb_2_C–MSNs–PEG, and Nb_2_C–MSNs–SNO groups, which indicated that the Nb_2_C–MSNs–SNO composite nanosheets were biocompatible and NIR‐II laser irradiation had no harm to cells (Figure 4f). Through semiquantitative analysis, it is found that the ratio of PI/calcein AM of the Nb_2_C–MSNs–PEG + laser and Nb_2_C–MSNs–SNO + laser groups was significantly higher than other four groups, and the ratio of the Nb_2_C–MSNs–SNO + laser group was significantly higher than the Nb_2_C–MSNs–PEG + laser group (Figure 4g), which also demonstrated that Nb_2_C–MSNs–SNO possessed high efficiency for killing cancer cells. Above results indicate the high promise of employing Nb_2_C–MSNs–SNO composite nanosheets as a nanoagent for synergistic cancer therapy via photonic thermogaseous therapeutic strategy.

### 2.4 In Vivo Toxicity, Blood Circulation, Biodistribution, and Metabolism of Nb_2_C–MSNs–SNO

The in vivo biocompatibility of Nb_2_C–MSNs–SNO composite nanosheets was investigated for guaranteeing potential further clinical transformation. Healthy Kunming mice were randomly divided into four groups, including control group and three treated groups with varied dosages ([Nb] = 5, 10, and 20 mg kg^−1^, respectively). The major organs and blood samples were collected at varied time intervals (1, 7, and 28 days) after postinjection. After blood tests and liver/kidney function evaluations, it has been found that there was no significant difference between the control group with treatment groups (Figure S12, Supporting Information). Subsequently, the hematoxylin and eosin (H&E) staining of major organs (Figures S13–S15, Supporting Information) shows no inflammatory cell infiltration, hemorrhage, and necrosis, demonstrating that these Nb_2_C–MSNs–SNO would not cause acute or chronic reactions in vivo. These results indicate that the fabricated Nb_2_C–MSNs–SNO composite nanosheets are featured with excellent biocompatibility and high potentials for clinical transformation. The half‐life of Nb_2_C–MSNs–SNO was calculated to be 1.28 h (Figure S16a, Supporting Information). Inductively coupled plasma emission spectrometry (ICP‐OES) was employed for quantitative analysis of Nb_2_C–MSNs–SNO biodistribution in vivo (Figure S16b, Supporting Information). The results exhibited that after intravenous injection of 12 h, around 3.23% of Nb was accumulated into tumor due to the enhanced permeability and retention (EPR) effect. In addition, the urine and feces contained 21.59% and 8.25% of Nb amount (Figure S17, Supporting Information), respectively, at 48 h after postinjection, which suggests that Nb_2_C–MSNs–SNO composite nanosheets were mainly excreted through the urinary system and digestive system, demonstrating the desirable excretion behavior of these composite nanosheets in vivo.

### 2.5 In Vitro and In Vivo PA Imaging

**Figure 5:**
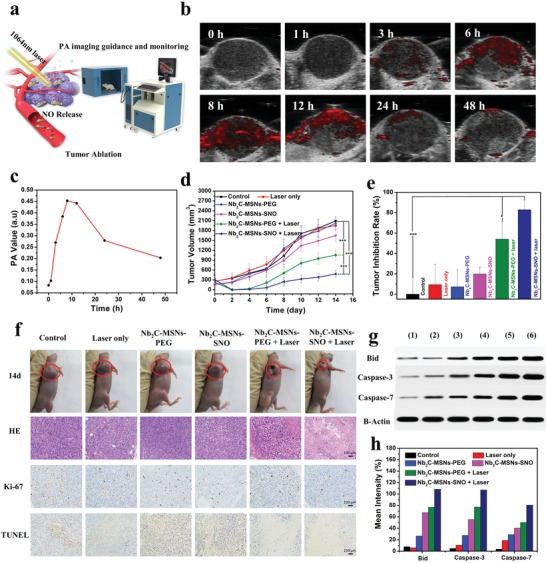
In vivo contrast‐enhanced PA imaging and synergistic photonic‐triggered theramogaseous therapy of tumor as enabled by Nb_2_C–MSNs–SNO composite nanosheets. a) Schematic illustration showing PA imaging after intravenous administration of Nb_2_C–MSNs–SNO composite nanosheets and the process of synergistic thermogaseous therapy of tumor in vivo. b) In vivo PA images and c) corresponding signal intensities of tumor after intravenous administration of Nb_2_C–MSNs–SNO at different time intervals (0, 1, 3, 6, 8, 12, 24, and 48 h). d) Time‐dependent tumor‐growth profiles after various treatments, including control, 1064 nm laser only, Nb_2_C–MSNs–PEG, Nb_2_C–MSNs–SNO, Nb_2_C–MSNs–PEG + 1064 nm laser, and Nb_2_C–MSNs–SNO + 1064 nm laser. (*P*‐values: * < 0.05, ** < 0.01, and *** < 0.001). e) Tumor‐inhibition ratio of 4T1‐tumor‐bearing mice at 14th day after different treatments (*P*‐values: * < 0.05, ** < 0.01, and *** < 0.001). f) Digital images of tumor regions of 4T1‐tumor‐bearing mice at the 14th day after different treatments. Histological assessments in tumor tissue after varied treatments, including H&E staining, TUNEL assay, and antigen Ki‐67 immunofluorescence staining. Scale bar: 100 µm. g) Western blot analysis of the Bid, caspase‐3, and caspase‐7 protein expression in tumor‐bearing mice with different treatments. h) Bid, caspase‐3, and caspase‐7 as quantified by normalized to the B‐actin control.

Since the Nb_2_C–MSNs–SNO composite nanosheets possess distinct NIR‐II absorbance, desired photothermal‐conversion efficiency in NIR‐II region, and superior photonic thermogaseous therapeutic effect in vitro, sequential/synergistic therapeutic efficacy in vivo with PA imaging guidance and monitoring was further evaluated (**Figure** 5a). The PA images and corresponding PA signal values illustrated that the PA signal of the Nb_2_C–MSNs–SNO exhibited a concentration‐dependent feature (Figure S18, Supporting Information), indicating that Nb_2_C–MSNs–SNO composite nanosheets could serve as a desirable PA contrast agent for potential guidance and monitoring of therapeutic process. The 4T1‐tumor‐bearing mice were intravenously administrated with Nb_2_C–MSNs–SNO composite nanosheets, and the PA imaging was conducted at varied time intervals, which revealed efficient tumor accumulation of Nb_2_C–MSNs–SNO and high tumor contrast of in vivo PA imaging. In addition, the PA signal was gradually elevated via a time‐dependent manner and reached the maximum at 8 h postinjection due to the accumulation of Nb_2_C–MSNs–SNO in the tumor site, which also suggested the optimal irradiation time after postinjection (Figure 5b,c). These results demonstrated that the designed Nb_2_C–MSNs–SNO composite nanosheets could act as an excellent PA contrast agent.

### 2.6 In Vivo Photonic Thermogaseous Cancer Therapy against Tumor‐Bearing Mice

Encouraged by the intriguing in vitro photonic thermogaseous therapeutic practices, sequential/synergistic therapeutic efficacy of Nb_2_C–MSNs–SNO was evaluated by adopting 4T1‐tumor‐bearing nude mice. The 4T1‐tumor‐bearing mice were divided into six groups (*n* = 5 in each group), including 1) control (treated with PBS), 2) PBS + 1064 nm laser irradiation (power density: 1.0 W cm^−2^), 3) Nb_2_C–MSNs–PEG ([Nb] = 10 mg kg^−1^), 4) Nb_2_C–MSNs–SNO ([Nb] = 10 mg kg^−1^), 5) Nb_2_C–MSNs–PEG + 1064 nm laser irradiation ([Nb] = 10 mg kg^−1^, power density: 1.0 W cm^−2^), and 6) Nb_2_C–MSNs–SNO + 1064 nm laser irradiation ([Nb] = 10 mg kg^−1^, power density: 1.0 W cm^−2^). After (2), (5), and (6) groups were exposed to 1064 nm laser irradiation for 10 min at the power density of 1.0 W cm^−2^, the tumor‐site temperature of (2) group mice treated with PBS increased from 30.4 to 38.4 °C, while the tumor‐site temperature of the (5) group treated with Nb_2_C–MSNs–PEG and the (6) group treated with Nb_2_C–MSNs–SNO increased from 29.9 to 50.3 °C and 30.3 to 51.8 °C, respectively (Figure S19, Supporting Information).

It is turned out that negligible weight fluctuations were recorded in all groups, demonstrating negligible systemic side effect of these different treatments (Figure S20, Supporting Information). In addition, major organs (heart, liver, spleen, lung, and kidney) were not observed obviously with inflammations or damages by H&E staining after different treatments, which further demonstrated that these treatments would not induce potential toxicities and side effects (Figure S21, Supporting Information). The tumor volumes were measured every 2 days, and corresponding tumor‐growth curves were recorded (Figure 5d). The results exhibited that significant growth of tumor volumes in the groups of control, laser only, Nb_2_C–MSNs‐PEG only, and Nb_2_C–MSNs–SNO only were observed, while the groups of Nb_2_C–MSNs–SNO upon 1064 nm laser irradiation (inhibition rate: 82.91%) (Figure 5e) showed enhanced tumor‐suppression efficacy as compared to the group of Nb_2_C–MSNs–PEG under laser irradiation (inhibition rate: 54.05%), verifying that Nb_2_C–MSNs–SNO possess excellent synergistic/sequential thermogaseous therapeutic efficacy. Although the group of Nb_2_C–MSNs–PEG upon 1064 nm laser irradiation exhibited obvious effect on inhibiting tumor growth by photonic hyperthermia, the best antitumor efficacy was acquired in the NO donor–assistant group (Nb_2_C–MSNs–SNO + 1064 nm laser) with both photonic ablation and gaseous therapies. The tumor weights at the end of treatments also confirmed a desirable synergistic efficacy (PTT and gas therapy) of Nb_2_C–MSNs–SNO against 4T1 cancer (Figure S22, Supporting Information).

In order to investigate the mechanism of photonic thermogaseous therapy with synergistic efficacy, tumor sections after various treatments were acquired from all groups of mice at 24 h after the treatments, which were analyzed by H&E, antigen Ki‐67, and terminal deoxynucleotidyl transferase‐mediated dUTP‐biotin nick end labeling (TUNEL) staining (Figure 5f). H&E and TUNEL staining images showed that the group of Nb_2_C–MSNs–SNO under 1064 nm laser irradiation possessed highest efficacy for killing 4T1 cancer cells in comparison to the groups of control, laser only, Nb_2_C–MSNs–PEG only, Nb_2_C–MSNs–SNO only, and Nb_2_C–MSNs–PEG with 1064 nm laser irradiation, which indicated effective cancer‐cell apoptosis induced by thermogaseous therapeutic modality. The proliferative levels of the 4T1 cancer cells were evaluated by antigen Ki‐67 staining, and remarkable suppression on cell proliferation was observed in groups (5) and (6), which matched well with the H&E and TUNEL staining results, further demonstrating that Nb_2_C–MSNs–SNO enabled high therapeutic efficacy. Additionally, tumor tissues were collected from all groups of mice at 24 h after various treatments and the expression levels of apoptosis proteins were detected (Bid, caspase‐3, and caspase‐7; Figure 5g,h). The results showed that the expressions of apoptotic proteins in groups (5) and (6) were significantly up regulated, but the protein expression levels of the (6) group were higher than the (5) group, while other groups of apoptosis proteins were at low expression levels. Bid protein and caspase‐3/caspase‐7 protein are important members of two families which are responsible for regulating the apoptosis process of cells, where caspase‐3 and caspase‐7 are the executioner subsets of a family of phylogenetically conserved heterodimeric cysteine proteases (caspases), and bid is a pro‐apoptotic protein in the Bcl‐2 family. These two families are linked by a mitochondrial apoptotic pathway. Once the pro‐apoptotic proteins of the Bcl‐2 family are activated, mitochondrial membrane potential will be changed, followed by cytochrome c (cyt c) releasing into the cytoplasm to form a complex termed apoptosome with Apaf‐1, dATP, etc. Then, caspase‐3/caspase‐7, as downstream executioner subsets of caspase family, would be activated to destroy cell structure and induce cell apoptosis.^33^ Above results suggest the efficient activation of the Bid, caspase‐3, and caspase‐7 of the Nb_2_C–MSNs–SNO via the developed photonic thermogaseous therapy, which could induce tumor‐cell apoptosis more efficiently.

## 3 Conclusion

In summary, we have established a novel therapeutic modality based on photo‐triggered thermogas‐generating nanoreactor for photonic thermogaseous cancer therapy. This photonic/thermal‐responsive nanoreactor can integrate multiple functions by engineering the Nb_2_C surface with mesoporous silica layer, where thermoresponsive NO donor (RSNO) was effectively loaded into mesoporous structure for “on‐demand” photothermal‐triggered NO generation. Once irradiated by an NIR‐II laser, the “core” of this nanoreactor can rapidly achieve photothermal transformation, which would further trigger the release of NO from encapsulated NO donor for thermogaseous therapy. Systematic in vitro and in vivo experiments have demonstrated that this nanoreactor exhibited intriguing therapeutic efficacy toward cancer cells via inducing cancer cells' apoptosis. Meanwhile, photoacoustic imaging could be used to potentially guide and monitor the therapeutic process, achieving the precise cancer treatment. Furthermore, in vivo and in vitro experiments demonstrated that the Nb_2_C–MSNs–SNO nanoreactor possessed superior biocompatibility, which laid a foundation for further potential clinical transformation. Therefore, this work not only provides a new efficient cancer‐therapeutic modality, but also pioneers a field of generating therapeutic gas by NIR‐II laser regulation for cancer therapeutics.

## Conflict of Interest

The authors declare no conflict of interest.

## Supporting information

Supporting Information
