# Supplementary material for: Nanomedicine‐Enabled Photonic Thermogaseous Cancer Therapy
Source: Adv Sci (Weinh). 2019 Nov 26;7(2):1901954. doi: 10.1002/advs.201901954 (PMC6974955; doi:10.1002/advs.201901954)
Supplement: Supplementary file 1 — Supporting Information [file ADVS-7-1901954-s001.pdf]

Copyright WILEY-VCH Verlag GmbH & Co. KGaA, 69469 Weinheim, Germany, 2019.

ADVANCED SCIENCE
Open Access

Supporting Information

for *Adv. Sci.,* DOI: 10.1002/advs.201901954

Nanomedicine-Enabled Photonic Thermogaseous Cancer Therapy

Haohao Yin, Xin Guan, Han Lin, Yinying Pu, Yan Fang, Wenwen Yue, Bangguo Zhou, Qiao Wang, Yu Chen,* and Huixiong Xu**

Copyright WILEY-VCH Verlag GmbH & Co. KGaA, 69469 Weinheim, Germany, 2019.

Supporting Information

Nanomedicine-Enabled Photonic Thermogaseous Cancer Therapy

Haohao Yin,[1] Xin Guan,[1] Han Lin,[2] Yinying Pu,[1] Yan Fang,[1] Wenwen Yue,[1] Bangguo Zhou,[1] Qiao Wang,[1] Yu Chen, [2]* and Huixiong Xu[1]**

A: Experimental Section

1. Materials

Cetyltrimethylammonium chloride solution (CTAC, 25 wt% in H_2O), 3-(triethoxysilyl)propyl methacrylate (MPTES) and tert-Butyl nitrite were purchased from Sigma-Aldrich (China). triethanolamine (TEA), ammonium hydroxide and Sodium chloride (NaCl) were obtained from Shanghai Sinopharm Chemical Reagents Co., Ltd. Tetraethyl orthosilicate (TEOS), methylbenzene and methanol were obtained from Shanghai Lingfeng Chemical Reagent Co., Ltd. Nb_2AlC powder and tetrapropylammonium hydroxide (TPAOH) were purchased from Forsman Scientific Co., Ltd. Hydrofluoric acid (HF, 50%) was obtained from Sinopharm Chemical Reagents Co., Ltd.

2. Characterization

Transmission electron microscopy (TEM) images were recorded on JEM-2100F transmission electron microscopy (TEM, 200 kV). Scanning transmission electron microscopy (STEM) and corresponding element mapping scans images were obtained by a field-emission Magellan 400 microscope (FEI Company). X-ray photoelectron spectroscopy (XPS) was applied on an ESCAlab250 (Thermal Scientific). Atomic force microscopy (AFM) images were acquired by Bruker Multi Mode system. Fourier transform infrared spectroscopy (FTIR) was used to analyze the chemical bonds of composite nanosheets. UV-vis NIR spectrums were tested on UV-3101PC Shimadzu spectroscope. The concentrations of Nb element were determined by inductively coupled plasma emission spectrometry (ICP-OES). The Zetasizer Nanoseries was applied to record the size and zeta potential of nanosheets. The thermal-field video and corresponding temperature detection were collected by use of an infrared thermal imaging instrument (FLIR A325SC camera). The confocal laser scanning microscopy (CLSM) images were acquired under FV1000 microscope (Olympus Company).

3. Synthesis of Nb_2C Nanosheets.

Two-dimensional Nb_2C nanosheets were synthesized by two-step exfoliation procedure. In order to remove the middle Al layer, HF (50 mL) was added into Nb_2AlC under stirring for 48 h at room temperature (RT). After centrifugation and washing with ethanol and water several times, the collection was dispersed in TPAOH (60 mL) with stirring for 72 h at RT. Then, the Nb_2C nanosheets were collected after centrifugation and washing with ethanol and water for several times.

4. Synthesis of Nb_2C -MSNs Composite Nanosheets.

CTAC (1 g) and TEA (0.01 g) solutions were diluted in deionized water (5 mL) with stirring for 20 min at RT. Followed by dropwise addition into Nb_2C aqueous solution (5 mg, 10 mL), and stirred and ultrasound treatment for 1.5 h at room temperature. TEOS (100 μL) was then dropwise added into the above mixture solution, and stirred for 1 h at 80°C in water bath to form the Nb_2C-MSNs structure. To collect Nb_2C-MSNs products, centrifugation and washing with deionized water for 3 times were sequentially conducted. To remove CTAC, as pore-forming agents, the above products were extracted with a mixture solution (50 mL, methanol: NaCl = 500 ml: 4 g) for 12 h at RT, then washed with ethanol for 3 times.

5. Surface PEGylation and NO Donor Conjugation of Nb_2C-MSNs.

To improve the dispersion and hydrophilicity of the nanosheets, mPEG-Silane (50 mg) was added into Nb_2C-MSNs aqueous solution (25 mL) and stirred for 24 h. The Nb_2C-MSNs-PEG were collected by centrifugation and washing with deionized water for 3 times to remove extra mPEG-Silane. In order to graft S-nitrosothiols (R-SNO) group into the mesoporous structure, Nb_2C-MSNs-PEG was initially modified with -SH group and then reacted with tert-butyl nitrite. Ammonia (30%, 100 µL) and MPTES (75 µL) were added into Nb_2C-MSNs-PEG solution (ethanol, 20 mL) and stirred for 12 h at RT. Then the products of Nb_2C-MSNs-SH were washed with ethanol for 3 times and dispersed in 15 ml mixture solution (methanol/toluene = 4: 1). Followed by adding t-butyl nitrite (1 mL) into above solution and stirring for 24 h in the dark, the resulting Nb_2C-MSNs-SNO products were collected by centrifugation and washed with deionized water for 3 times.

6. *In Vitro* photothermal Performance of Nb_2C-MSNs-SNO.

The infrared thermal imager was applied to record the temperature changes during the laser irradiation (1064 nm) and determine the *in vitro* photothermal performance of Nb_2C-MSNs-SNO. Firstly, the extinction coefficient and photothermal-conversion efficiency of

Nb_2C-MSNs-SNO were determined. To record temperature-changes curves of Nb_2C-MSNs-SNO, different concentrations of Nb_2C-MSNs-SNO aqueous solutions ([Nb] = 0, 10, 20, 40, 80, 160, 320 μg mL^{-1}) under NIR-II laser irradiation at different power densities (0.5, 0.75, 1.0, 1.25, and 1.5 W cm^{-2}) for 10 min. Subsequently, in order to evaluate the photothermal stability of Nb_2C-MSNs-SNO composite nanosheets, temperature changes of Nb_2C-MSNs-SNO solution ([Nb] = 80 μg mL^{-1}) through five laser on/off cycles (1.5 W cm^{-2}) were recorded.

7. Measurement of NO Release.

The NO release from Nb_2C-MSNs-SNO was qualitatively assessed and quantitatively measured using a typical Griess assay. To determine the cumulative release of NO from Nb_2C-MSNs-SNO after different treatments, including different irradiation power densities (0, 0.5, 1.0, 1.5 and 2.0 cm^{-2}) and varied concentrations ([Nb] = 0, 6.25, 12.5, 25, 50, 100 and 150 μg mL^{-1}), the Griess agent was added into different groups and recorded by microplate reader. The fluorogenic probe, 3-Amino,4-aminomethyl-2’,7’-difluorescein diacetate (DAF-FM DA), was applied to verify the intracellular NO release. 4T1 cells were pre-seeded into CLSM-specific dishes at a density of 1 × 10^5 and cultured for 12 h. After addition of Nb_2C-MSNs-SNO at the concentration of 50 μg mL^{-1} and co-incubation for 12 h, the cells were incubated with DAF-FM DA (50 μM) for 20 min, and then exposed to 1064 nm laser at different power densities (0, 0.5, 1, 1.5, 2.0 W cm^{-2}). The CLSM images of released NO were captured by excitation at 488 nm.

8. CLSM Analysis and Flow Cytometry Observation of Intracellular Endocytosis.

4T1 cells were seeded into CLSM-specific culture dishes (35 mm × 10 mm) and 6-well plates at a density of 1 × 10^5 and incubated for 24 h at 37 °C, following the medium was replaced by FITC-loaded Nb_2C-MSNs-SNO (1 mL, [Nb] = 50 μg mL^{-1}), which was then co-incubated for 0, 1, 2, 4, and 8 h. Then, the medium was washed with PBS for 3 times, followed by cell nucleus was stained by DAPI for 20 min. CLSM imaging experiments were carried out on an Olympus FV1000 laser-scanning microscope equipped with a CW NIR laser (λ = 980 nm) as the excitation source. The flow cytometry was then used to evaluate intracellular endocytosis. Moreover, the mechanism of cellular uptake was investigated by pre-treatments of MβCD, sucrose, and amiloride for 30 min, followed by incubation with FITC-loaded Nb_2C-MSNs-SNO (1 mL, [Nb] = 50 μg mL^{-1}) for 4 h. To further quantify the intracellular fluorescence intensity, all cells were collected, and the fluorescence signals were measured.

9. *In Vitro* Cytotoxicity Assay.

4T1 cells and HUVEC were incubated in DMEM medium (high glucose, GIBCO, Invitrogen) with 10% fetal bovine serum (FBS) and 1% penicillin/streptomycin under 5% CO_2 atmosphere at 37 °C. For *in vitro* cytotoxicity assay of Nb_2C-MSNs-PEG and Nb_2C-MSNs-SNO, the 4T1 cells and HUVEC were seeded in 96-well plates at 1×10^4 cells/well for 24 h to allow cells to attach. Varied concentrations ([Nb] = 0, 12, 25, 50, 100, and 200 μg mL^{-1}) of Nb_2C-MSNs-PEG and Nb_2C-MSNs-SNO were added into the cells and co-incubated for 24 h. The following cells viability were determined by CCK-8 (Cell Counting Kit, Beyotime Institute of Biotechnology, Shanghai, China).

10. *In Vitro* Synergistic therapy Effect of 4T1 Cells.

The 4T1 cells were seeded in 96-well plates at a density of 1×10^4 cells/well for 12 h to attach on the plates, then coincubated with Nb_2C-MSNs-PEG and Nb_2C-MSNs-SNO at varied concentrations ([Nb] = 0, 12.5, 25, 50, 100, 200 μg mL^{-1}) for 4 h. Following, these cells were exposed to 1064 nm laser irradiation for 5 min at 1.0 W cm^{-2}. In addition, to evaluate the cells viability after irradiation at different power densities (0, 0.5, 1.0, 1.5, and 2.0 W cm^{-2}), 4T1 cells were coincubated with Nb_2C-MSNs-PEG and Nb_2C-MSNs-SNO at same concentrations for 5 min, then these cells were irradiated for 5 min using 1064 nm laser at different power densities. Finally, a standard CCK-8 protocol was used to assess cell viabilities.

11. *In Vitro* Flow Cytometry and CLSM Observation of Synergistic Therapies Effect of Nb2C-MSNs-SNO.

Flow cytometry was applied to quantitatively assess cell apoptosis levels. Typically, 4T1 cells were seeded into 6-well plates for 24 h to attach on the dishes, then the medium was replaced by PBS, Nb_2C-MSNs-PEG and Nb_2C-MSNs-SNO ([Nb] = 100 μg mL^{-1}) and incubated for 8 h. These cells were irradiated for 5 min using 1064 nm laser at different power densities (0, 0.5, 1.0 and 1.5 W cm^{-2}). The cells were collected by dissociation *via* trypsin, centrifugation and washing for 3 times with PBS. Finally, the mixture solution, containing 5 μL PI and 5 μL FITC, was added into these cells for 20 min incubation. The flow cytometry was then used to evaluate cell-apoptosis levels. 4T1 cells were pre-seeded into CLSM-specific dishes at a density of 1×10^5 and cultured for 12 h to attach on the plates. Then, the PBS, Nb_2C-MSNs-PEG and Nb_2C-MSNs-SNO ([Nb] = 100 μg mL^{-1}) were co-incubated with 4T1 cells for 4 h. Then, these cells were irradiated for 5 min using 1064 nm laser at different power densities (0, 0.5, 1.0 and 1.5 W cm^{-2}). After treatments, 4T1 cells were observed by CLSM, in which live cells and dead cells were stained by Calcein-AM and PI, respectively.

12. *In Vivo* Toxicity Assay.

Animal experiment procedures were confirmed to the guidelines for the Animal Care Ethics Commission of Shanghai Tenth People's Hospital, Tongji University School of Medicine. Forty-eight healthy female Kunming mice (~ 20 g) were randomly separated into 4 groups (*n* = 12) and then intravenously administered with PBS, Nb_2C-MSNs-SNO ([Nb] = 5 mg kg^{-1}), Nb_2C-MSNs-SNO ([Nb] = 10 mg kg^{-1}) and Nb_2C-MSNs-SNO ([Nb] = 20 mg kg^{-1}), which were then fed for varied days (1, 7 and 28 d) to evaluate *in vivo* toxicity. The blood samples were collected for serum biochemistry assays and complete blood panel tests, including red blood cells (RBC), white blood cells (WBC), platelets (PLT), mean corpuscular volume (MCV), hemoglobin (HGB), mean corpuscular hemoglobin (MCH), mean corpuscular hemoglobin concentration (MCHC), and albumin (ALB), total protein (TP), globulin (GLB), blood urea nitrogen (BUN), creatinine (CREA), total bilirubin (TBIL), alanine transaminase (ALT), and aspartate transaminase (AST). The major organs (heart, liver, spleen, lung and kidney) were obtained, fixed in 10% paraformaldehyde, and stained with hematoxylin and eosin (H&E) for histological analysis.

13. *In Vivo* Blood Circulation, Biodistribution and Metabolism of Nb_2C-MSNs-SNO.

For pharmacokinetic analysis, five healthy female Kunming mice were intravenously administered with Nb_2C-MSNs-SNO, followed by 10 μl blood samples were collected and immersed into saline (1 mL) containing heparin sodium (50 unit mL^{-1}) at varied time points (1, 5, 10, 15, and 30 min, 1, 2, 4, 8, 10, and 24 h). The Nb amounts of these samples were determined by ICP-OES. For biodistribution analysis, 4T1 tumor-bearing mice were randomly divided into 6 groups (*n* = 3) when the tumor volume reached around 150 ~ 200 mm^3 and then intravenously administered with Nb_2C-MSNs-SNO. Then, the tumors and major organs (heart, liver, spleen, lung and kidney) were collected at different time points (1, 2, 4, 8, 12, and 24 h) of post-injection, following these tumor and organs were weighed and homogenized. ICP-OES was used to determine the Nb amount in these organs/tissues. The metabolism process of Nb_2C-MSNs-SNO was assessed in female Kunming mice (*n* = 4), Nb_2C-MSNs-SNO in PBS ([Nb] = 5 mg kg^{-1}) was intravenously administered into the mice. The faeces and urine were collected at varied time points (2, 6, 12, 24, 36, and 48 h) and the Nb content in faeces and urine was determined by ICP-OES.

14. *In Vitro* and *in Vivo* PA Imaging.

For *in vitro* imaging, the PA images and corresponding signal values of varied concentrations ([Nb] = 62.5, 125, 250, 500 and 1000 μg mL^{-1}) of Nb_2C-MSNs-SNO

nanosheets were acquired by VisualSonics. For *in vivo* PA imaging, the 4T1 tumor-bearing mice were anesthetized and intravenously administered with Nb_2C-MSNs-SNO solution (200 µL, [Nb] = 1000 µg mL^{-1}) when the tumor volume reached around 150 mm^3, followed by the PA imaging. The corresponding signal values at varied time points (0, 1, 3, 6, 8, 12, 24 and 48 h) were recorded by VisualSonics.

15. *In Vivo* Photonic Thermogaseous Cancer Therapy against Tumor-Bearing Mice.

To establish xenograft tumor model, 4T1 cells (1×10^6 cell/site) dispersed in saline solution (100 µL for each mouse) were injected into the back of mice, which were the healthy female Balb/c nude mice (5 weeks old). These mice were randomly divided into six groups (n = 5), including (1) control (treated with PBS), (2) PBS + 1064 nm laser irradiation (Power density: 1.0 W cm^{-2}), (3) Nb_2C-MSNs-PEG ([Nb] = 10 mg kg^{-1}), (4) Nb_2C-MSNs-SNO ([Nb] = 10 mg kg^{-1}), (5) Nb_2C-MSNs-PEG + 1064 nm laser irradiation ([Nb] = 10 mg kg^{-1}, Power density: 1.0 W cm^{-2}) and (6) Nb_2C-MSNs-SNO + 1064 nm laser irradiation ([Nb] = 10 mg kg^{-1}, Power density: 1.0 W cm^{-2}). The volume of the tumors and weight of the mice were measured every 2 days during 14 days period. The tumor volume was measured according to the following formula: (tumor length) × (tumor width)^2 /2. The tumors weights were determined in 14-day period after synergistic therapy. Subsequently, the tumors and major organs (heart, liver, spleen, lung and kidney) were sliced and stained with H&E, TUNEL and Ki-67 for histological analysis. Furthermore, to understand the mechanism of tumor growth inhibition, the apoptotic proteins (Bid, Caspase-3, Caspase-7) expression levels were tested.

16. Statistical Analysis.

All data were expressed as mean ± standard deviation (SD). Student's t test was applied to evaluate the date with different significance levels (P values: *<0.05, **<0.01, ***< 0.001).

B: Supplementary figures

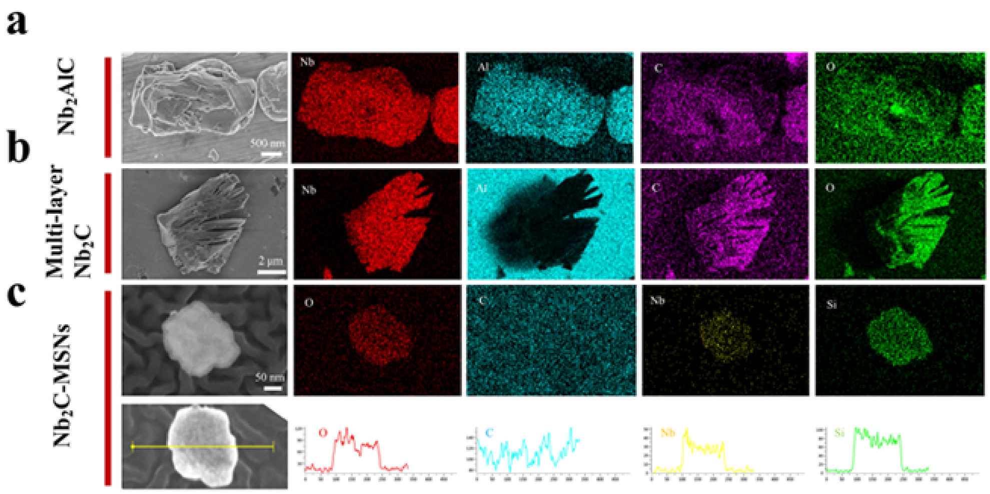

Figure S1. (a) SEM and corresponding elemental mapping (Nb, Al, C and O) for original Nb_2AlC crystallites. (b) SEM and corresponding elemental mapping (Nb, Al, C and O Elemental mapping for HF-etched Nb_2AlC (multilayer Nb_2C). (c) Elemental mapping lateral (upper images) and corresponding element-linear scanning (vertical (down image) of Nb_2C-MSNs composite nanosheets (Nb, Si, C and O).

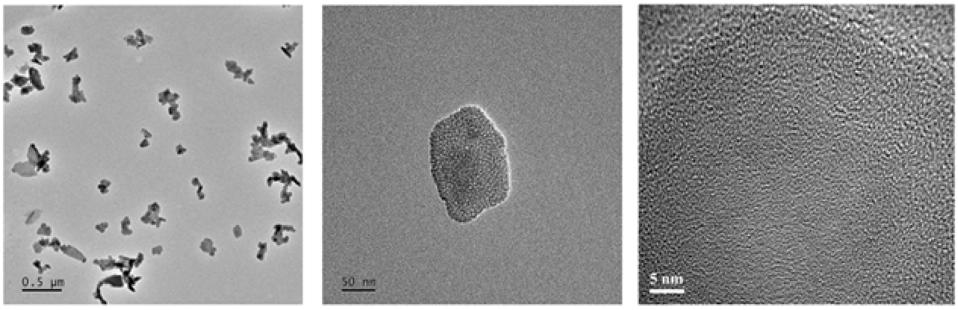

Figure S2. TEM images of Nb_2C-MSNs composite nanosheets at different magnifications.

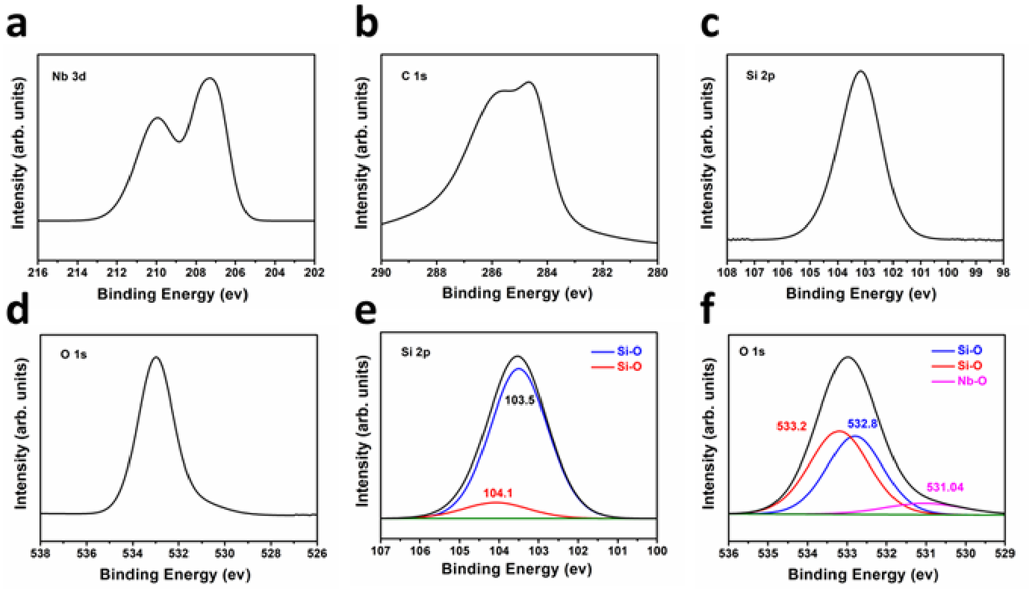

Figure S3. XPS spectra of Nb_2C-MSNs composite nanosheets in (a) Nb 3d, (b) C 1s, (c) Si 2p, and (d) O 1s regions. (e) Si 2p and (f) O 1s core level spectra of Nb_2C-MSNs composite nanosheets

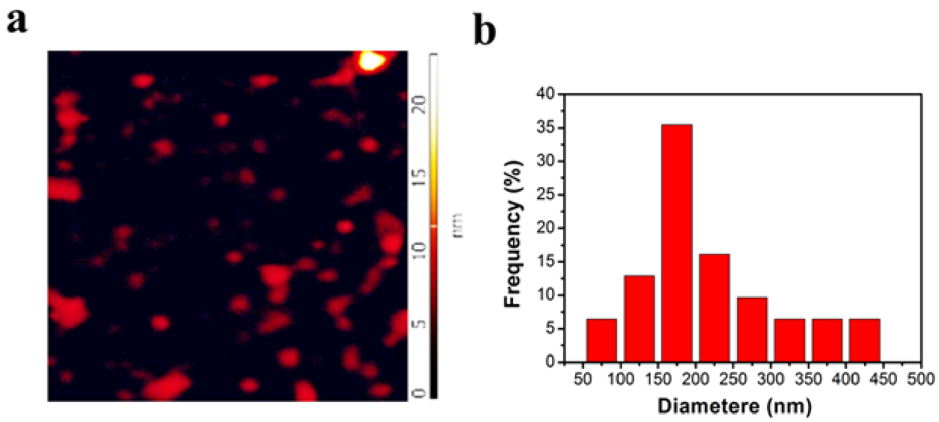

Figure S4. (a) AFM image of Nb_2C nanosheets. (b) Lateral size distribution analysis of Nb_2C nanosheets.

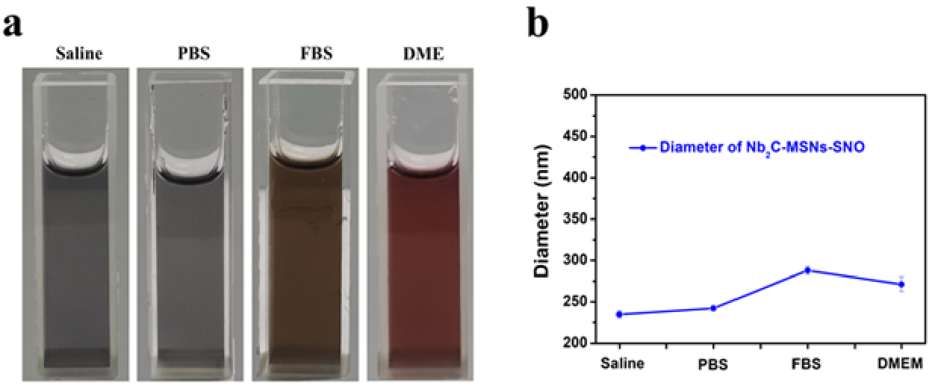

Figure S5. (a) Digital images and (b) hydrodynamic sizes of Nb_2C-MSNs-SNO composite nanosheets dispersed in a variety of simulated physiological media (saline, PBS, FBS, and DMEM).

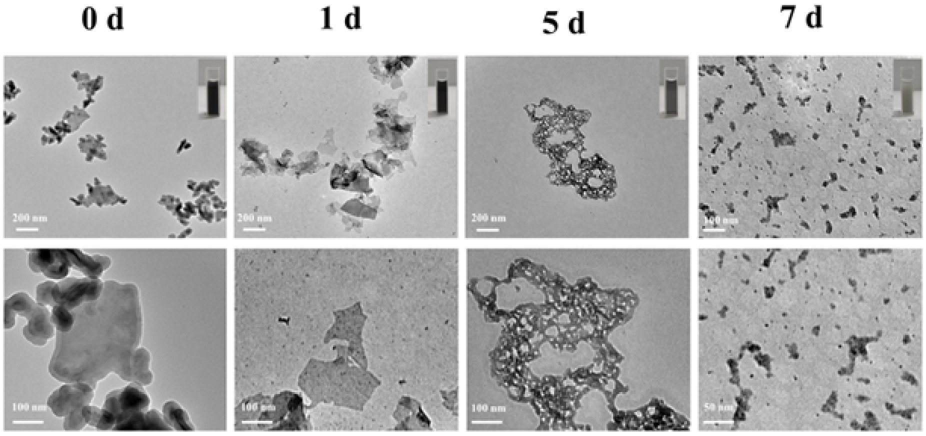

Figure S6. TEM images of Nb_2C-MSNs-SNO composite nanosheets dispersed in PBS for varied time intervals of degradation treatment (0, 1, 5, and 7 days). The down images were the magnification images of the top pictures. Insert are the corresponding digital images.

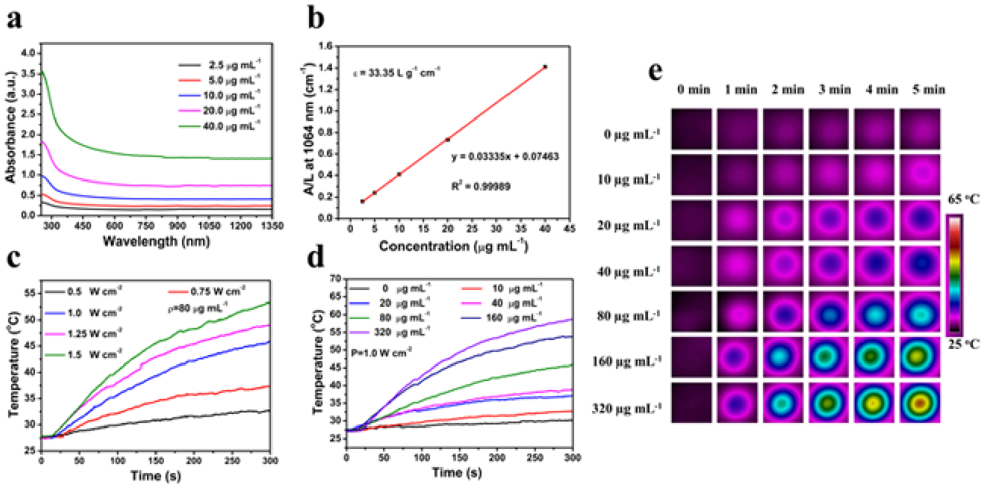

Figure S7. (a) UV–vis spectra of Nb_2C-MSNs-SNO composite nanosheets at different concentrations ([Nb] = 2.5, 5, 10, 20, 40 μg mL^{-1}). (b) Mass extinction coefficient of Nb_2C-MSNs-SNO at 1064 nm (NIR-II). (c) Photothermal-heating curves of Nb_2C-MSNs-SNO at elevated power densities under 1064 nm laser irradiation ([Nb] = 80 μg mL^{-1}). (d) Photothermal-heating curves and (e) corresponding thermal images of Nb_2C-MSNs-SNO at different concentrations ([Nb] = 0, 10, 20, 40, 80, 160, 320 μg mL^{-1}) under 1064 nm laser irradiation.

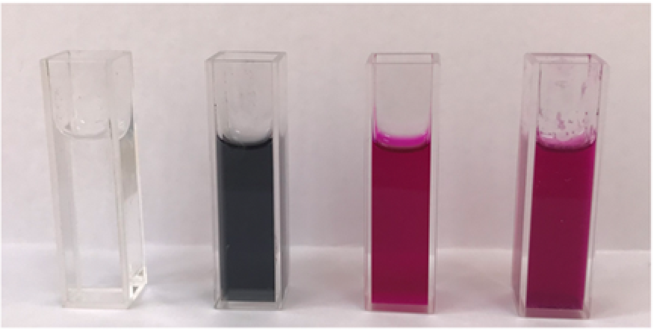

Figure S8. Qualitative evaluation of NO release from negative control group (deionized water), positive control group ($NaNO_2$), Nb_2C-MSNs-SNO and Nb_2C-MSNs-PEG after exposure to 1064 nm laser irradiation.

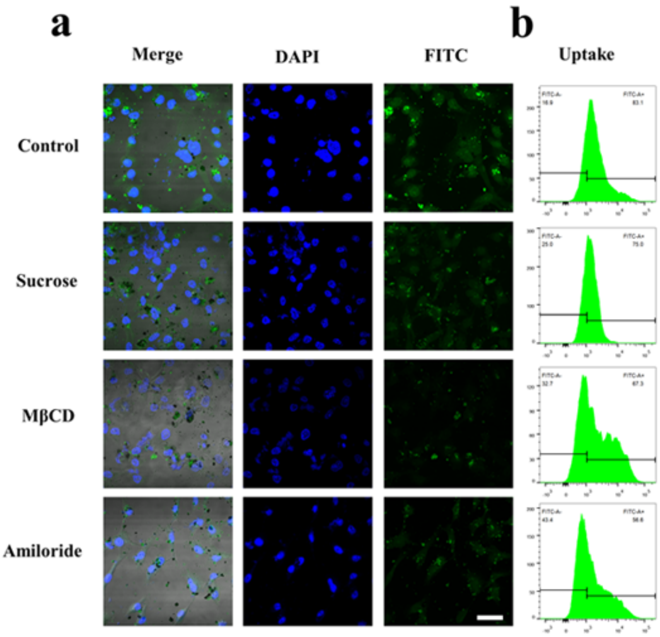

Figure S9. (a) CLSM images and (b) the corresponding flow cytometry analysis of cellular uptake of Nb_2C-MSNs-SNO composite nanosheets by 4T1 cancer-cell line after coincubation with different inhibitors. (scale bar: 20 μm)

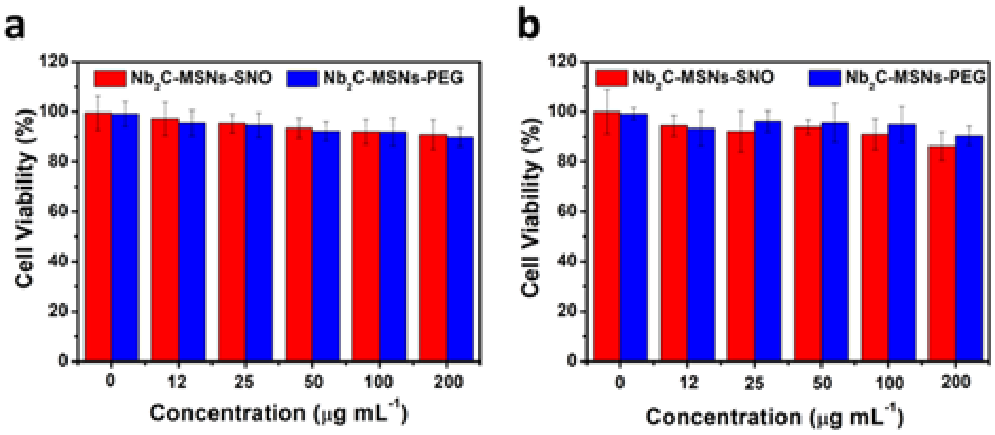

Figure S10. (a) Relative viabilities of 4T1 cells after being co-incubated with varied concentrations ([Nb] = 0, 12, 25, 50, 100, and 200 μg mL^{-1}) of Nb_2C-MSNs-PEG and Nb_2C-MSNs-SNO composite nanosheets. (b) Relative viabilities of HUVEC cells after being co-incubated with varied concentrations ([Nb] = 0, 12, 25, 50, 100, and 200 μg mL^{-1}) of Nb_2C-MSNs-PEG and Nb_2C-MSNs-SNO composite nanosheets.

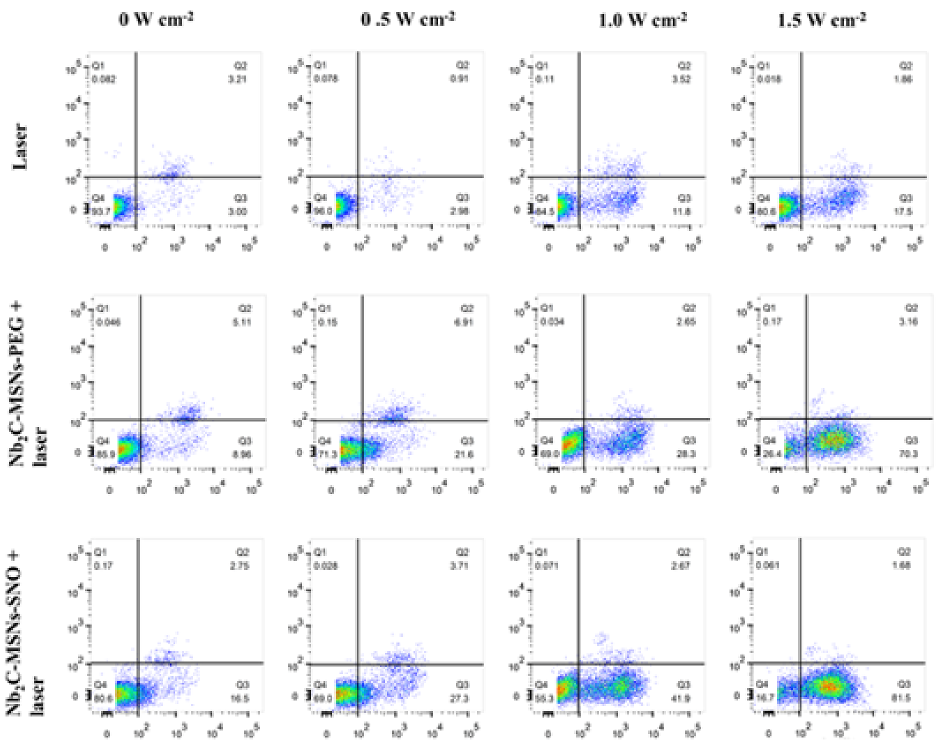

Figure S11. Flow cytometry quantification assay on relative viabilities of 4T1 cell after incubation with PBS, Nb_2C-MSNs-PEG and Nb_2C-MSNs-SNO composite nanosheets under different NIR-II laser power densities (0, 0.5, 1.0 and 1.5 W cm^{-2}).

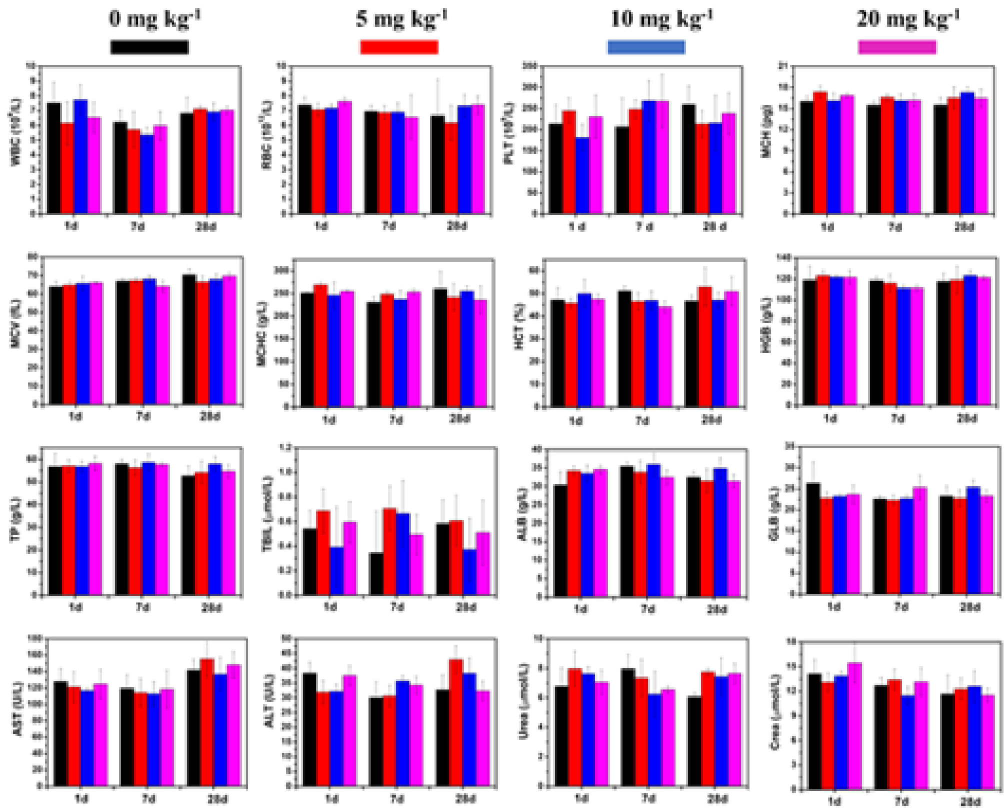

Figure S12. Hematological index and biochemical blood analysis of the mice with injection of different doses ([Nb] = 0, 5, 10, 20 mg kg^{-1}) of Nb_2C-MSNs-SNO for 1, 7, and 28 days feedings. The results include white blood cells (WBC), red blood cells (RBC), platelets (PLT), mean corpuscular hemoglobin (MCH), mean corpuscular volume (MCV), mean corpuscular hemoglobin concentration (MCHC), hematocrit (HCT), hemoglobin (HGB), total protein (TP), total ilirubin (TBIL), albumin (ALB), globulin (GLB), aspartate transaminase (AST), alanine aminotransferase (ALT), urea and creatinine (Crea).

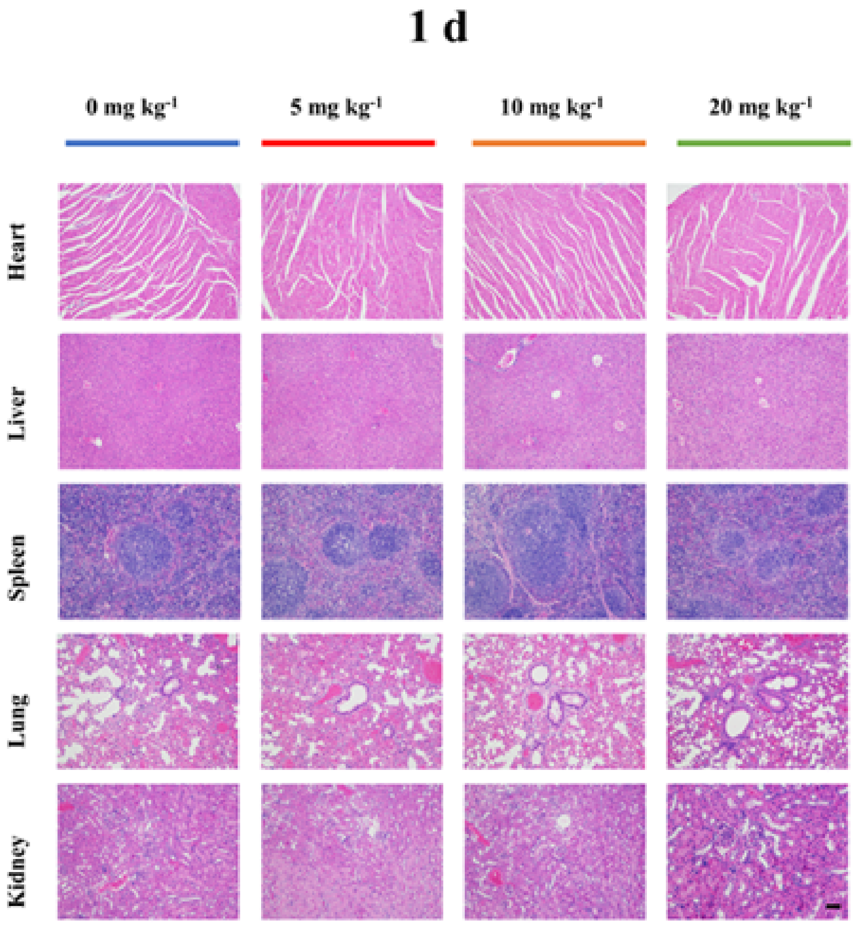

Figure S13. H&E stained images of the major organs (heart, liver, spleen, lung, and kidney) of the Nb_2C-MSNs-SNO-treated mice at the first day injected with varied doses of the composite nanosheets (scale bar: 100 μm).

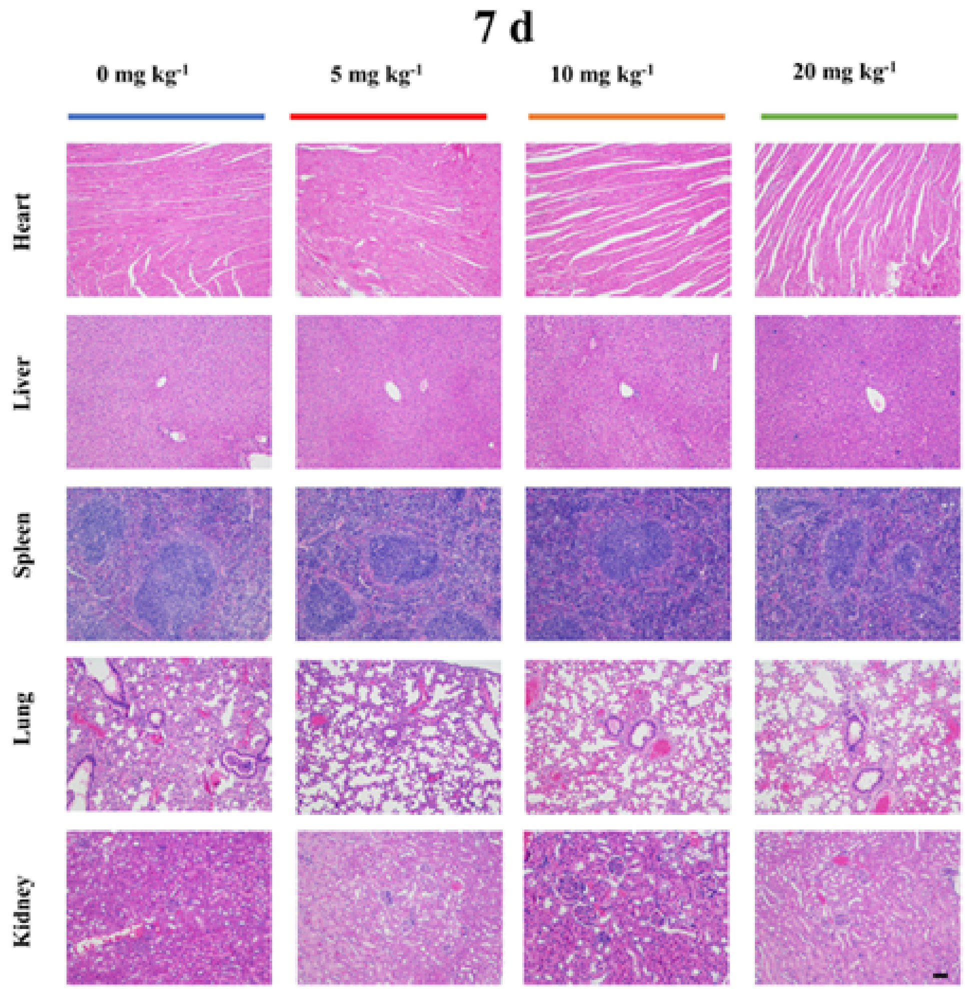

Figure S14. H&E stained images of the major organs (heart, liver, spleen, lung, and kidney) of the Nb_2C-MSNs-SNO-treated mice at the 7th day injected with varied doses of the composite nanosheets (scale bar: 100 μm).

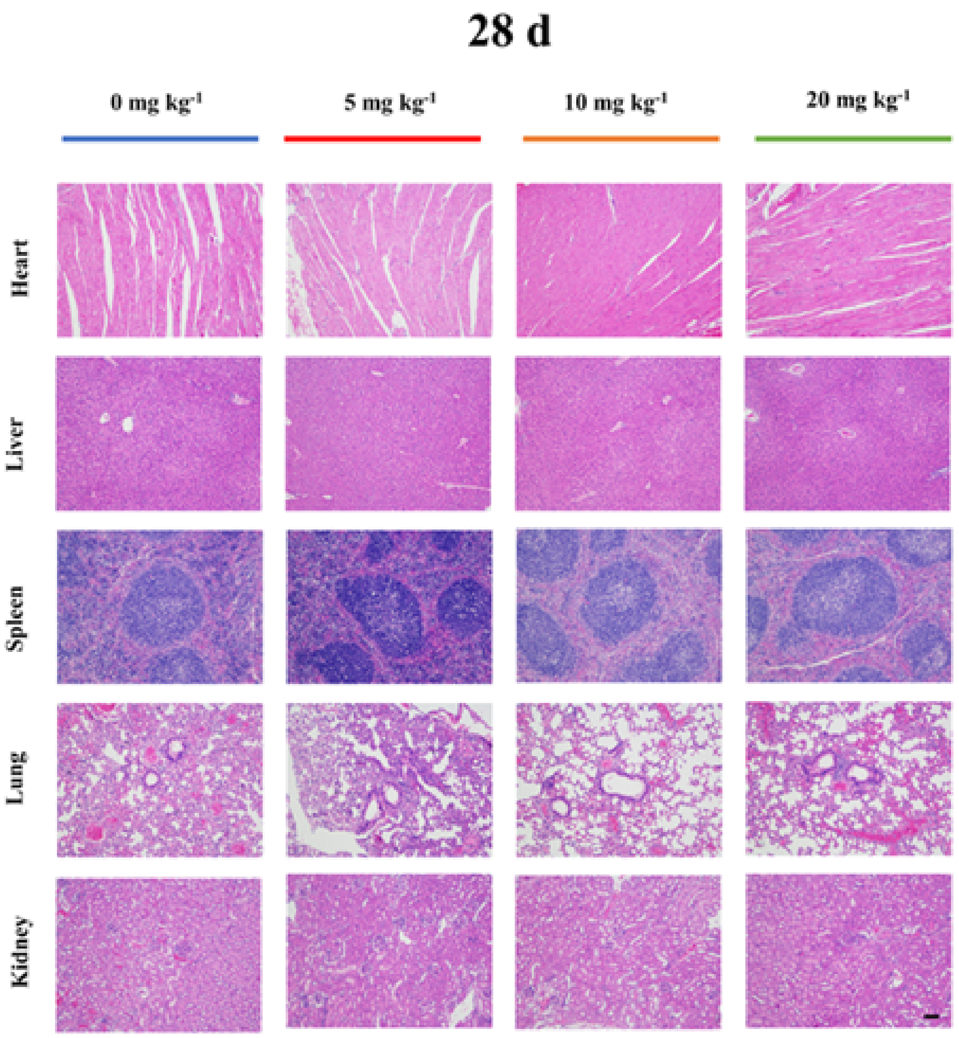

Figure S15. H&E stained images of the major organs (heart, liver, spleen, lung, and kidney) of the Nb_2C-MSNs-SNO-treated mice at the 28th day injected with varied doses of the composite nanosheets (scale bar: 100 μm).

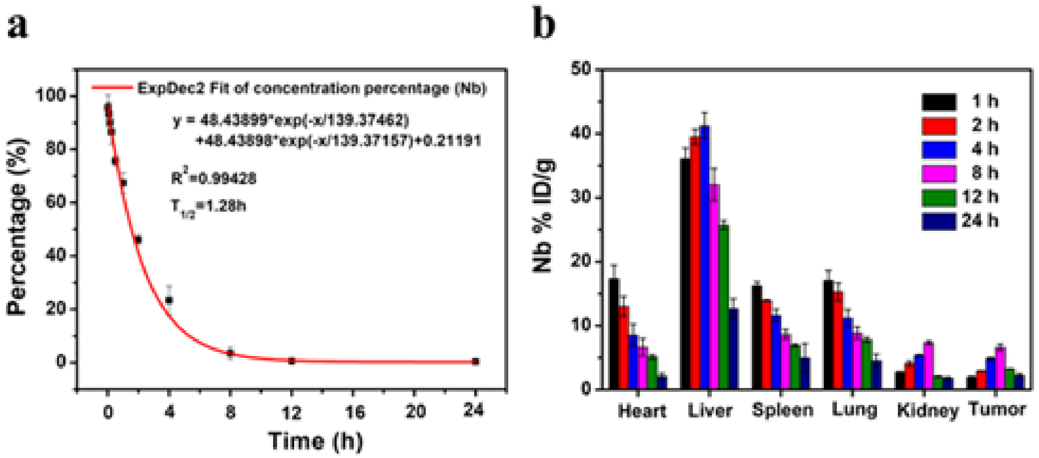

Figure S16. (a) Blood-circulation lifetime of Nb_2C-MSNs-SNO after injection into mice (n = 5). (b) Biodistribution of Nb (% ID of Nb per gram of tissues) in major organs and tumor after injection of Nb_2C-MSNs-SNO solution for varied time intervals (1, 2, 4, 8, 12 and 24 h, n = 3).

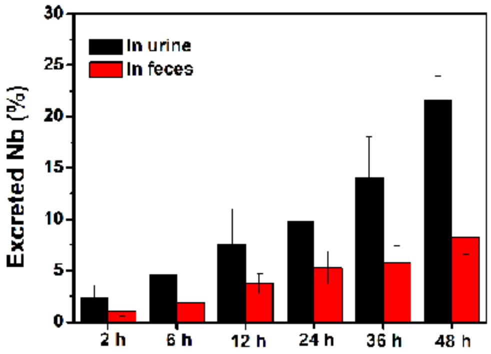

Figure S17. Cumulative excretion of Nb (in feces and urine) out of the mice (n = 4) body after injection of Nb_2C-MSNs-SNO for different time intervals (2, 6, 12, 24, 36 and 48 h).

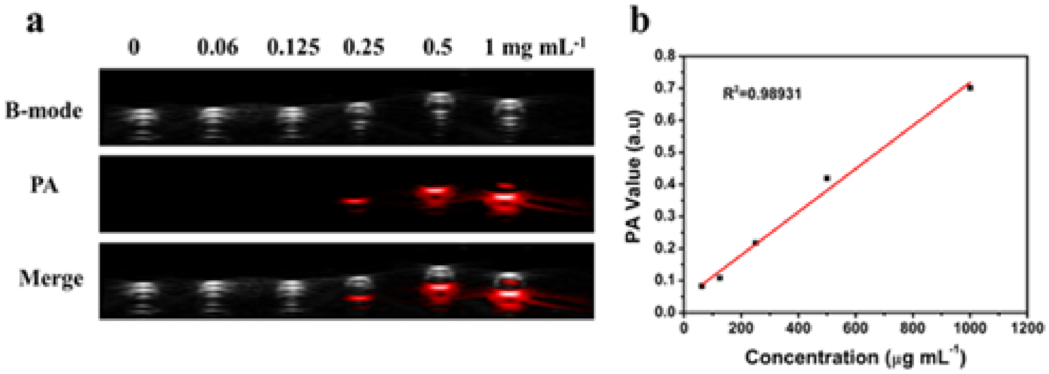

Figure S18. (a) *in vitro* PA images of Nb_2C-MSNs-SNO at different concentrations ([Nb] = 0, 0.06, 0.125, 0.25, 0.5 and 1 mg mL^{-1}) and (b) Quantitative curve of PA values.

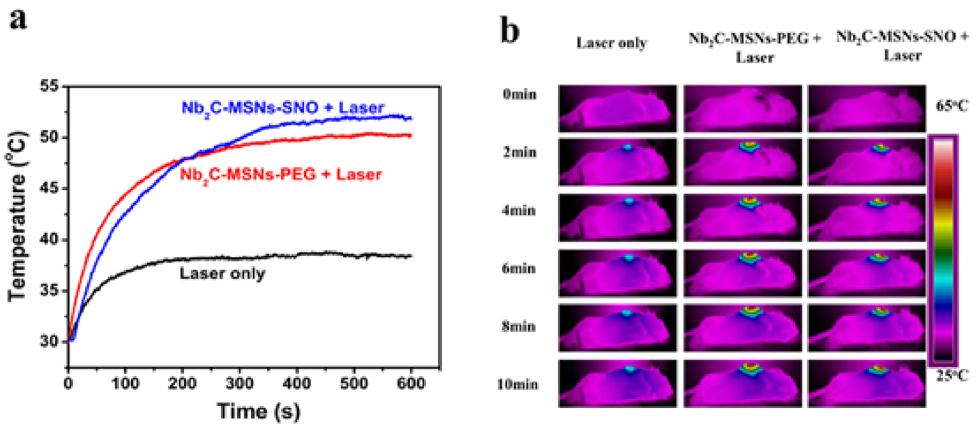

Figure S19. (a) Temperature-elevation curves and (b) corresponding IR images at the tumor areas of 4T1-tumor-bearing mice during different treatments, including 1064 nm laser only, Nb_2C-MSNs-PEG +1064 nm laser and Nb_2C-MSNs-SNO + 1064 nm.

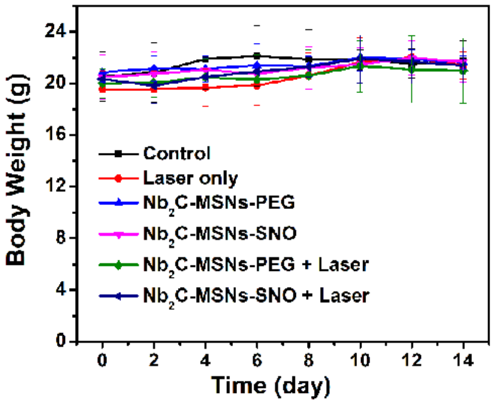

Figure S20. Time-dependent body-weight curves of nude mice (n = 5, mean ± SD) after various treatments, including control, 1064 nm laser only, Nb_2C-MSNs-PEG, Nb_2C-MSNs-SNO, Nb_2C-MSNs-PEG + 1064 nm laser and Nb_2C-MSNs-SNO + 1064 nm laser.

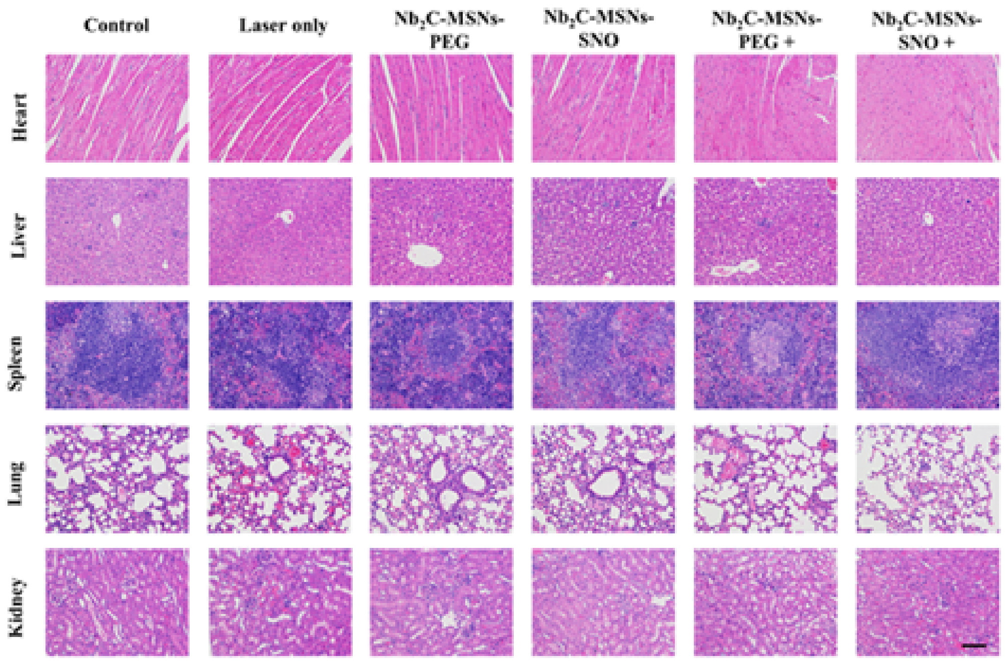

Figure S21. H&E-stained tissue sections of major organs (heart, liver, spleen, lung, and kidney) from mice with various treatments (scale bar: 100 μm).

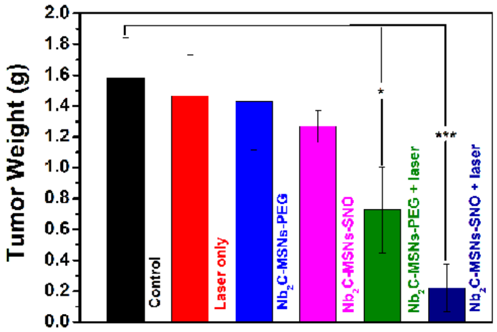

Figure S22. Tumor weights of 4T1-tumor-bearing mice at 14th d after different treatments (P values: * < 0.05, ** < 0.01, and *** < 0.001).
